# Infrastructure Availability for the Care of Congenital Heart Disease Patients and Its Influence on Case Volume, Complexity and Access Among Healthcare Institutions in 17 Middle-Income Countries

**DOI:** 10.5334/gh.968

**Published:** 2021-10-21

**Authors:** Isaac Wamala, Russell Gongwer, Kaitlin Doherty-Schmeck, Maria Jorina, Anne Betzner, Bistra Zheleva, Kimberlee Gauvreau, Christopher W. Baird, Kathy Jenkins

**Affiliations:** 1 Department of Cardiothoracic and Vascular Surgery, German Heart Institute Berlin, Berlin, DE; 2 Department of Cardiovascular Surgery, Charité Universitätsmedizin, Berlin, DE; 3 Department of Cardiac Surgery, Boston Children’s Hospital, Boston, US; 4 Center for Applied Pediatric Quality Analytics, Boston Children’s Hospital, Boston, US; 5 HealthCore, Inc., Watertown, US; 6 Center for Patient Safety Research and Practice, Brigham and Women’s Hospital, Boston, US; 7 Children’s Heart Link, Minneapolis, Minnesota, US; 8 Department of Cardiology, Boston Children’s Hospital, Boston, US; 9 Harvard Medical School, US

**Keywords:** Congenital Heart Surgery, Infrastructure, Surgical Capacity

## Abstract

The care for patients with congenital heart disease (CHD) is multi-disciplinary and resource intensive. There is limited information about the infrastructure available among programs that care for CHD patients in low and middle-income countries (LMIC). A survey covering the entire care-pathway for CHD, from initial assessment to inpatient care and outpatient follow-up, was administered to institutions participating in the International Quality Improvement Collaborative for Congenital Heart Disease (IQIC). Surgical case complexity-mix was collected from the IQIC registry and estimated surgical capacity requirement was based on population data. The statistical association of selected infrastructure with case volume, case-complexity and percentage of estimated case-burden actually treated, was analyzed. Thirty-seven healthcare institutions in seventeen countries with median annual surgical volume of 361 (41–3503) operations completed the survey. There was a median of two (1–16) operating room/s (OR), nine (2–80) intensive care unit (ICU) beds, three (1–20) cardiac surgeons, five (3–30) OR nurses, four (2–35) anesthesiologists, four (1–25) perfusionists, 28 (5–194) ICU nurses, six (0–30) cardiologists and three (1–15) interventional cardiologists. Higher surgical volume was associated with higher OR availability (p = 0.007), number of surgeons (p = 0.002), OR nurses (0.008), anesthesiologists (p = 0.04), perfusionists (p = 0.001), ICU nurses (p < 0.001), years of experience of the most senior surgeon (p = 0.03) or cardiologist (p = 0.05), and ICU bed capacity (p = 0.001). Location in an upper-middle income country (P = 0.04), OR availability (p = 0.02), and number of cardiologists (p = 0.004) were associated with performing a higher percentage of complex cases. This study demonstrates an overall deficit in the infrastructure available for the care of CHD patients among the participating institutions. While there is considerable variation across institutions surveyed, deficits in infrastructure that requires long-term investment like operating rooms, intensive care capacity, and availability of trained staff, are associated with reduced surgical capacity and access to CHD care.

## Background

The global prevalence of congenital heart disease (CHD) at birth is currently estimated to be 1.8 cases per 100 live births [1]. Moderate and severe forms of CHD affect approximately six of every one thousand babies born alive [2]. In 2017, CHDs led to an estimated 261,247 deaths, and a total of 589,479 years lived with disability globally and represent an increasingly prominent cause of infant and child mortality [1], especially in low and middle-income countries(LMIC) [1,3]. Advances in medical technology and knowledge over the past fifty years now allow the possibility to either correct or palliate the majority of congenital heart anomalies leading to improved length and quality of life for those affected [4,5,6].

A large disparity however exists in the global capacity to care for these patients. Four point eight billion of the world’s population lack access to safe and affordable surgical care [7]. The majority of this population live in LMIC [7,8]. The barriers to safe surgery in LMICs are overwhelmingly linked to resource availability [9]. It is estimated that there are less than two operating rooms for all forms of surgery per 100,000 people in low-income countries compared to more than 14 operating rooms per 100,000 people in high-income countries [10]. A substantial unmet need for cardiac surgery affects not only low-income but also middle-income countries [11,12,13,14]. In a 2018 study, low-income Mozambique had 0.1 cardiac centers per 15 million people with an estimated 5,800 patients per surgeon. There was a large variation among the surveyed middle-income countries, ranging from 0.08 centers per 13 million people in lower-middle income Nigeria to 1.6 centers per 0.6 million people in upper middle income Brazil [13]. Moreover, the provision of cardiac surgery in middle-income countries does not perfectly correlate with per capita gross domestic product or health related expenditure and is not evenly distributed between urban and rural areas [13].

The timely and effective care of CHD patients goes well beyond surgery alone. Successful outcomes require a skilled, dedicated multi-disciplinary team, based in a well-resourced integrated healthcare institution, working closely with the parents of the patients. Recommendations in high-income countries regarding the optimal resources for CHD-care [15,16,17] may not necessarily be relevant in LMIC.

The literature to date regarding the infrastructure available for the care of CHD patients in resource limited settings can be grouped into studies that have provided detailed information about one or a few localities, or those that have focused on a specific aspect of CHD-care such as surgery [18,19,20,21]. There lacks a study with detailed information covering all aspects of CHD care on a global scale.

To help reduce that gap, we aimed to assess the infrastructure availability across the entire hospital care pathway from initial patient evaluation, inpatient care to outpatient follow-up by designing a comprehensive survey based on present-day pediatric cardiac hospital care infrastructure evidence and recommendations. Between November 2016 and May 2017, we sent the survey to institutions enrolled in the International Quality Improvement Collaborative for Congenital Heart Disease (IQIC). The IQIC was formed to provide benchmarking data for congenital heart surgery in the developing world, with the overall goal of guiding quality improvement efforts and reducing mortality for congenital heart disease [22]. The IQIC database maintains audited data of case volume, complexity and outcomes at participating sites.

We conducted an across-the-board evaluation of available infrastructure among participating institutions, in order to explore how resource availability and spending priorities are translated into actual treatment capacity. In the interest of brevity, this report focusses on the institution-wide infrastructure and their influence on surgical capacity. Further details regarding special aspects like the cardiac catheterization, adult CHD and costs of care, and the influence of infrastructure on patient-level outcomes will be reported separately. We also discuss the wider public health implications of the findings in middle-income countries.

## Methodology

### Study design and participants

Infrastructure availability and total surgical volume were assessed using a detailed survey covering the entire in-hospital care pathway among institutions participating in IQIC. Surgical case complexity-mix data from the IQIC outcomes database for 2016 were used. An estimate of the required surgical capacity at the participating centers was made based on population data of the countries or regions for 2016. All of the 42 healthcare institutions that were active in the IQIC at the time of the survey were invited to participate. The Institutional Review Board at Boston Children’s Hospital reviewed and approved the study (IRB-P00022012).

### Patient and Public Involvement

It was not appropriate or possible to involve patients or the public in the design, or conduct, or reporting, or dissemination plans of this study.

### Survey tool and administration

In consultation with specialist cardiovascular health practitioners, a comprehensive survey tool was developed to assess the essential infrastructure for CHD-care available at the participating institutions. The survey covered the care pathway from screening, initial assessment, inpatient care to follow-up (Supplementary file). The survey comprised of 10 sections covering the operating room (OR), catheterization laboratory, ICU, step-down ward, regular ward, cardiovascular imaging, post-operative follow-up, adult congenital cardiac disease, multi-disciplinary care, staffing, and overall administrative characteristics. Each of these sections included both multiple response items and open-ended questions. The survey tool was piloted at three of the sites and modifications made accordingly. The health facilities had three months to complete the survey. Following survey completion and initial review by the survey staff, e-mail communication with the respondents was used to clarify any ambiguous answers or seek further information in case of incomplete responses. Further, the number of other alternative facilities with an active CHD care program in each country or region was assessed based on direct information from the participating institutions, supplemented by internet search and review of recent literature.

A final check of the survey data for quality and completeness was done before data analysis.

### IQIC Registry

The IQIC registry includes data about CHD surgeries at participating institutions, including type of surgery and the risk adjustment for congenital heart surgery (RACHS-1) [23] category. The RACHS-1 categories are a consensus-based method of risk adjustment for in-hospital mortality among children younger than 18 years after surgery for congenital heart disease. The IQIC registry records outcomes for in-hospital and 30-day mortality, and major infections following CHD surgery. A 10% random audit is performed for key variables on an annual basis.

### Population data

Population and crude birth rate data for the year 2016 was collected from the World Bank open data website for 15 countries. For China and India where the population exceeds a billion people, regional data was used instead of national data in order to facilitate a closer estimate of the local need in the areas served. For China, regional data for 2016 was collected from the 2018 national statistical yearbook the National Bureau of Statistics website and for India from the open data government platform as well as regional government websites [24].

### Analysis and Statistics

Survey responses were summarized at the institution level using frequencies and percentages for categorical variables, and medians and ranges for continuous variables unless otherwise noted. Stratification was done by program location and size (Supplementary file – Tables 3–6). For reporting, some categorical responses were condensed to create new categories (Supplementary Tables 2, 5–7). For questions where ‘Other’ was chosen as a category and details given, we either recoded the responses into one of the existing categories or created a new category based on the judgment of the analysis team. Open-ended questions were categorized into thematic topics whose frequencies were tabulated.

Exploratory statistical analyses were conducted to investigate the association of selected infrastructure with surgical volume, case complexity mix, and the proportion of cases performed to estimated new case burden were performed. For this analysis, surgical volume was taken as the total volume of CHD surgeries in children and adults as well as the number of non-CHD cardiac surgeries in children. The case complexity mix was taken as the percentage RACHS 3–6 cases of CHD surgeries in children. The approximate required surgical capacity was calculated by estimating the new case burden for CHD for each institution for the year 2016 as follows (Supplementary Table 3). The number of live births was calculated based on the most current known birthrate and the population of that year. Using a uniform estimated annual incidence of 6 per 1000 live births of moderate or severe CHD, the estimated number of new patients with moderate or severe CHD in each country or region was calculated. Based on the number of centers in the country (or region) known to provide CHD care, and assuming that they shared the surgical burden approximately equally, a calculation was made of how much of the national/regional surgical burden the participating programs would be expected to cover. Statistical comparisons were made using Fisher’s exact test, the Wilcoxon rank sum test, the Kruskal-Wallis test, or the Spearman rank correlation coefficient, as appropriate.

## Results

### Participating healthcare sites and demographics

Thirty-seven institutions in seventeen countries completed the survey (Supplementary Table 1). There were 13 (35%) institutions in the Americas, 18 (49%) in Asia and 6 (16%) in Eastern Europe. In 2016, 17 (46%) respondents were in World Bank lower-middle-income countries and 20 (56%) upper-middle-income countries. Four (11%) respondents were the only institution providing CHD-care in the country. Twenty-four (65%) institutions were public, 7 (19%) run by non-profit organizations (NPOs), and 6 (16%) were private. Twenty-eight (76%) institutions were associated with a medical school, 26 (70%) with a nursing school, and 31 (84%) with a cardiac surgery training program. The median estimated percentage of patients seeking surgical care at the facilities travelled the following distances; 30% (10–75%) travelled less than 50 km; 30% (10–60%) travelled 50 to 200 km; 20% (0–60%) from 200 to 500 km; a median of 10% (0–90%) travelled more than 500 km.

### Case volume and complexity-mix

In addition to CHD surgery, thirty-one (84%) programs also perform surgery for acquired heart disease such as rheumatic heart disease (RHD) in children. Twenty-eight (76%) institutions also perform CHD surgery on adults. The mean total surgical volume (CHD cases in children and adults plus non-CHD cases in children) was 361 (41–3503) per year. Based on case volumes the programs were stratified into 13 (35%) small to medium size programs performing less than 250 cases/year, 9 (24%) large programs with 250 to 500 cases a year and 15 (41%) very large programs where more than 500 cases per year are done. Case complexity according to RACHS-1 category was only available in 34 sites who participated in the IQIC registry in 2016. The median percentage of complex cases (RACHS Score 3–6) was 30%, with a wide range among respondents (0% to 60%).

### Estimated new CHD case requirement

The median estimated new CHD case burden at the participating sites was 472 (130–7057). The median proportion of cases performed to estimated new case burden was 1.0 (0.04–1.07). Fourteen (37%) programs performed less than half of estimated new CHD case burden (Supplementary Table 4).

### Operating room infrastructure, anesthesia, and disposable OR supplies

Infrastructure availability for the OR and anesthesia is summarized in supplementary table 1. The institutions had a median of two (2–4) cardiopulmonary bypass (CPB). While defibrillators were available in the majority, only 18 (51%) programs reported having rapid-fibrillators available. In 21 (59%) of the programs, intra-operative neurological monitoring is routinely conducted whereby eight (22%) of the institutions used brain symmetry index (BSI) and 13 (35%) programs used near infrared monitoring. Co-oximeters were routinely available and had adequate cartridges in 29 (78%) institutions. Fifteen (41%) programs use the alphastat and 21 (57%) the PHstat strategy for pH monitoring. Modified Ultrafiltration (MUF) is used in 32 (86%) programs.

Seventeen (46%) respondents at least sometimes resterilize and re-use items normally meant for single use. Items reported to be resterilized and reused included vascular patch materials, vascular conduits as well as cannulation suction tubing (web extra material).

### Medication, Prosthetic Materials and Blood Product Availability

**Figure 1:**
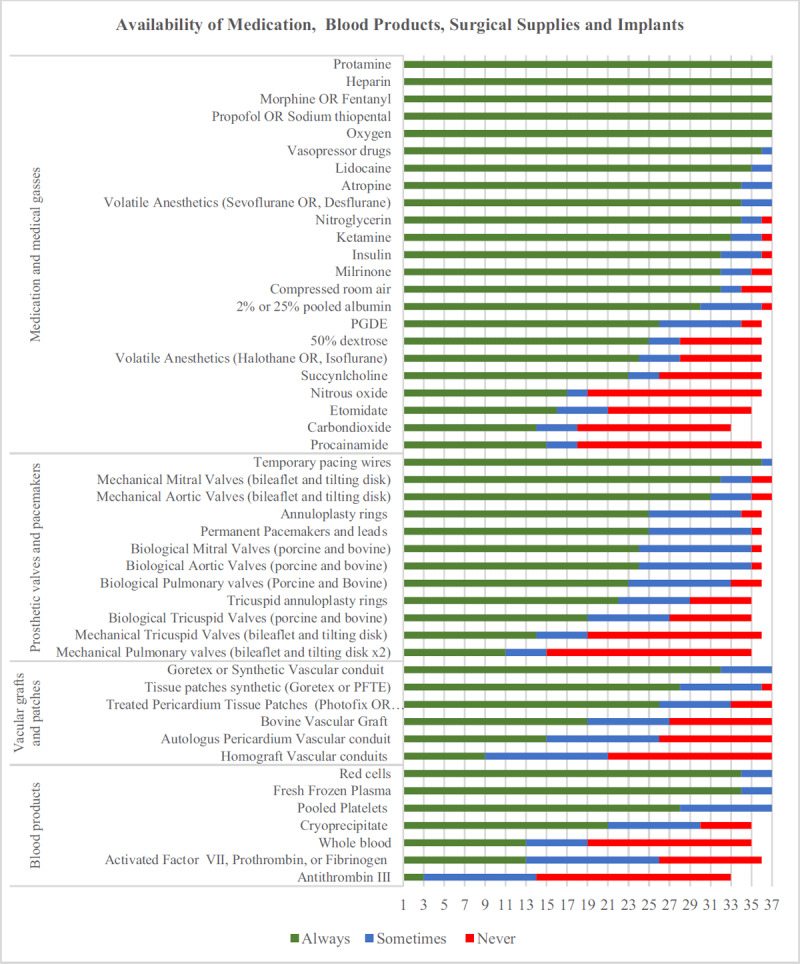
Availability of medication, blood products, surgical supplies and implants.

As summarized in Figure 1, essential medications and supplies like heparin, protamine, oxygen, propofol or thiopental, ketamine, vasopressors, lidocaine, atropine, temporary pacing wires, and red blood cells were usually available. Notably only 72%, 67% and 24% of institutions reported ready availability of prostaglandin E (PGDE), volatile anesthetics, and homografts respectively.

### Pre and Post-operative in-patient care

Infrastructure for in-patient care is summarized in Table 1. Twenty-two (60%) programs had a dedicated ICU for pediatric cardiac patients. Median ICU bed capacity was nine (2–80) with a median typical occupancy of three (1–45). The person at the attending physician or consultant level primarily responsible for ICU management of post-operative CHD patients was a cardiac intensivist in 28 (76%) institutions.

**Table 1:** Infrastructure for pre and post-operative in-patient care.

Infrastructure Variable		Facilities (N = 37)

ICU availability for paediatric CHD patients	Paediatric cardiac dedicated Shared with adult Shared with other paediatric	22 (60%) 12 (32%) 3 (8%)
ICU availability for adult CHD pts	Adult CHD Pts. go to paediatric cardiac ICU; Adult CT ICU; Other	17 (46%) 15 (41%) 3 (13%)
Neonatal ICU (NICU) available		14 (38%)
Ventilators for Neonates available		36 (97%)
High Frequency Oscillators available		20 (54%)
Incubators available		29 (78%)
Warming tables (N = 36)		30 (83%)
Dedicated code cart (N = 36)		33 (92%)
Emergency code cart fully stocked with all necessary medications		33 (89%)
		
Paediatric formulations of medication available for code cart	All code carts; Some; None	18 (55%) 8 (24%) 7 (21%)
		
Sterile emergency instruments available in ICU		30 (81%)
ECMO/or MCS programme		20 (54%)
Attending surgeon usually present for patient transport		28 (76%)
Formal multidisciplinary handover from OR to ICU regularly done		33 (89%)
Attending level physician responsible for post-op ICU management of CHD patients	Cardiac Intensivist General Intensivist Cardiac Surgeon Paediatrician Neonatologist	28 (76%) 3 (8%) 2 (5%) 2 (5%) 2 (5%)
Formal ICU Rounds regularly conducted at least once daily Dedicated CHD Care coordinators available		37 (100%) 16 (43%)
Social workers available		24 (65%)
Multidisciplinary case meetings/or rounds	Regular Scheduled Multi-Disciplinary Rounds	18 (49%)
	Multi-Disciplinary Rounds/ Meetings as needed No Regular Multi-Disciplinary rounds	17 (46%) 2 (5%)
An intermediate care ward		18 (50%)

For ICU care of neonatal patients, 14 (38%) programs had a separate Neonatal ICU (NICU). A catheterization lab was on site in 97% of the programs while only 20 (54%) of programs had a functioning extra corporeal membrane oxygenation (ECMO) or mechanical circulatory support (MCS) program.

Half (50%) of programs have an intermediate care ward, whereby the median capacity was seven (3–36) beds and the median typical occupancy was three (1–27) beds. The median capacity of the general care ward for CHD patients was 18 (4–85) beds.

Sixteen programs (43%) have dedicated care coordinators to facilitate the multi-disciplinary care. Twenty-four programs (65%) have dedicated social workers. In 18 institutions (49%), scheduled multi-disciplinary rounds or meetings are regularly conducted. A formal checklist is routinely used during transfer of patients from ICU to the ward in 23 (62%) of programs.

### Imaging

Echocardiography within an hour of an emergency was available in 34 (92%) programs. Ninety-seven percent of programs had the capability to perform sedated echocardiography. Computer tomography, magnetic resonance imaging and nuclear imaging were available in 81%, 54% and 46% of surveyed programs.

### Screening, access to care and post-hospital follow-up

Prenatal screening in the institution’s catchment area was always or often performed in 17 (46%) institutions. In suspected CHD cases following screening, fetal echocardiography was performed often or always-in 28 (76%), institutions. Newborn children were often or always screened for CHD in 18 (49%) institutions. For newborn screening for CHD, clinical examination was reported in 29 (78%), pulse oximetry in 30 (81%) and echocardiography in six (16%) institutions. Nineteen (51%) programs perform community screening for CHD among infants and children.

Time between referral and evaluation by a pediatric cardiologist was 24 hours in 15 (41%), within one week in 13 (35%) and longer than one week in nine (24%) institutions. The time between initial evaluation and first intervention is less than two days in 26 (70%), three to seven days in seven (19%) and greater than one week in four (11%) programs. In nine (24%) institutions, it takes less than a week between referral and surgery for elective cases. In 11 (30%) programs, it takes one to four weeks, one to three months in 10 (27%) programs and longer than three months in six (16%) programs.

Thirty (81%) programs reported a follow-up of greater than 75% of patients for six weeks after surgery. Only 10 (27%) programs reported having greater than 75% follow-up or ongoing care for pediatric patients and eight (21%) programs for adult patients longer than one year. Long distance from the hospital was the main barrier to follow-up in 21 (57%) institutions. Other factors included patient families not understanding the need in five (14%) and patients following up elsewhere in 10 (27%).

### Core Infrastructure and systems

In 34 (92%) institutions, main power supply was via a national electrical grid while in three (8%) it was generators. Thirty-five (95%) programs have backup power supply available. The backup power supply covered the entire hospital in 28 (76%) programs and in six (16%) programs was reserved for critical areas. Power surge protectors were installed in 33 (89%) institutions.

Five (13%) programs used a purely electronic medical record system, eight (22%) used a paper-based system and 24 (65%) used a mixed system. Doctors’ orders were entered electronically in 15 (41%) programs, written on paper in 21 (57% of programs) and one program used a mixed system.

### Staffing

There was a median of three (1–20) cardiac surgeons, five (0–30) OR nurses, four (2–35) anesthesiologists, four (1–25) perfusionists, six (0–20) ICU physicians, 28 (5–194) ICU nurses, nine (2–80) regular ward nurses, three (0–30) respiratory therapists, six (0–30) cardiologists and three (1–15) interventional cardiologists. The number of programs where the most senior specialist physician had more than 10 years of experience was 29 (78%) in surgery, 28 (76%) in cardiology, 29 (78%) in interventional cardiology, 31 (84%) in cardiac anesthesia and 21 (57%) in intensive care medicine. The median number of physicians who had completed formal training and certification in their area of specialization was three (1–20) in pediatric cardiac surgery, two (0–20) in anesthesia, two (0–15) in interventional cardiology and five (0–30) in cardiology. In 30 (81%) institutions, most or all perfusionists had completed formal training and certification in perfusion. Seventeen institutions (46%) received periodic staff reinforcements through visiting cardiac surgical teams from abroad. The teams visited a median of two (1–11) times in a year.

### Self-expressed Limitations

**Figure 2:**
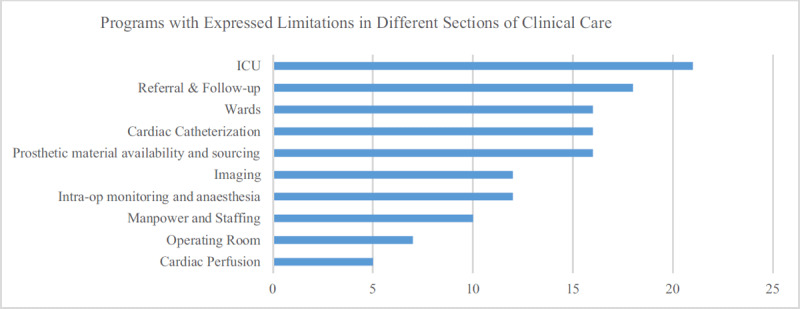
Number of programs that expressed limitations in specific areas of clinical care.

**Figure 3:**
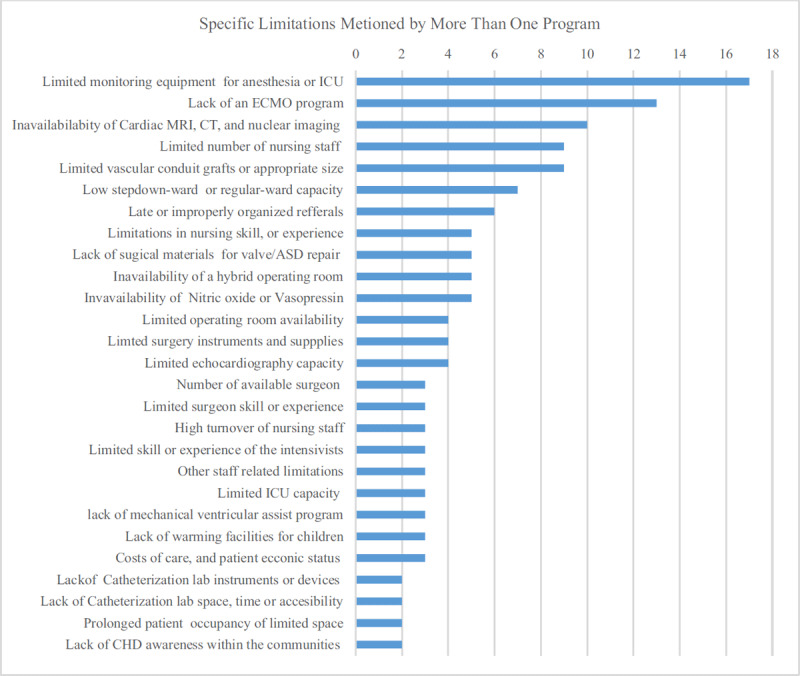
Aggregated limitation themes mentioned as free text.

Limitations in the intensive care unit, in the patient referral and follow-up system, the general wards, the catheterization laboratory and in prosthetic material availability and sourcing were mentioned by at least one third of the programs (Figure 2) as factors affecting their ability to perform optimal CHD care. In open-ended entries, limitations in the monitoring equipment for anesthesia or ICU, the lack of an ECMO program, unavailability of cardiac imaging modalities like MRI, CT-Scan, nuclear imaging as well as the limited staffing number were the most frequent themes mentioned (Figure 3).

### Stratification and associations

Tables showing infrastructure availability stratified by program size and geography are included in the Supplementary Tables 2 and 5–7. In comparing public to private and NPO run institutions, there was no significant difference in terms of case volume (p = 0.46), case complexity mix (p = 0.48), OR availability (p = 1), ICU capacity (p = 0.25), number of surgeons (p = 1), operating room nurses (p = 0.38), anesthetists (p = 1) among the surveyed programs. Private hospitals had significantly more cardiologist (p = 0.013).

Higher surgical volume was associated with higher OR availability (p = 0.007), number of surgeons (p = 0.002), OR nurses (p = 0.008), anesthesiologists (p = 0.04), perfusionists (p < 0.001), ICU nurses (p < 0.001), years of experience of the most senior surgeon (p = 0.03) or cardiologist (p = 0.05), and ICU bed capacity (p < 0.001) (Table 2).

**Table 2:** Association of case volume and case complexity mix with selected infrastructure.

Variable	Case Volume (N = 37)			Case Complexity Mix (N = 34)		

	Number of Sites	Median [IQR] number of surgical cases per year	P-value/or Spearman r (P-value)	Number of Sites	Median [IQR] Percent of Cases with RACS 3–6	P-value/or Spearman r (P-value)

Country Income Group 2018			0.17			0.04
Upper Middle	20	272 [165, 569]		20	37 [28,45]	
Lower Middle	17	381 [293, 680]		14	24 [20,31]	
Type of Facility			0.46			0.48
Public	24	381 [207, 760]		22	31 [20,45]	
Private	6	264 [170, 361]		5	34 [24,37]	
NPO	7	369 [261, 530]		7	30 [0,37]	
Associated with Medical School			0.21			0.67
Yes	28	311 [207, 622]		26	31 [20,41]	
No	9	530 [361, 1126]		8	34 [21, 46]	
Operating room availability			0.007			0.02
CHD dedicated, or shared but with ≥3 ORs	26	455 [261, 688]		23	37 [24, 45]	
OR Shared and 1 or 2 ORs	11	210 [101, 339]		11	23 [17, 30]	
CPB machine availability			0.11			0.54
CHD dedicated, or shared but with ≥3 machines	26	365 [234, 759]		23	31 [21, 42]	
CPB Shared and 1 or 2 machines	11	261 [113, 529]		11	30 [7, 48]	
Availability of bypass packs, cardioplegia tubing, arterial and venous cannula			0.33			0.38
Always (for all 3)	31	369 [210, 688]		30	32 [21, 42]	
Not always	6	272 [170, 548]		4	23 [13, 36]	
Number of surgeons			0.49 (0.002)			0.18 (0.31)
1–2	8	219 [112, 277]		8	22 [18, 44]	
3–4	15	380 [195, 653]		14	30 [21, 37]	
≥5	14	560 [339, 1057]		12	34 [27, 47]	
Years of experience, most senior surgeon			0.03			0.25
≤10	8	213 [124, 248]		7	25 [19, 30]	
>10	29	381 [282, 680]		27	34 [20, 43]	
Number of OR nurses			0.44 (0.008)			0.12 (0.51)
<4	10	205 [110, 328]		10	36 [26, 45]	
4–8	15	381 [204, 673]		13	25 [18, 41]	
≥9	11	380 [339, 760]		10	34 [23, 38]	
Number of cardiac anaesthesiologists			0.34 (0.04)			0.28 (0.11)
<4	16	277 [200, 517]		15	26 [18, 42]	
4–6	12	375 [222, 635]		10	31 [21, 45]	
≥7	9	530 [282, 1428]		9	34 [30, 37]	
Number of perfusionists			0.53 (<0.001)			0.14 (0.44)
<4	16	237 [200, 381]		15	30 [19, 41]	
4–5	14	350 [170, 680]		12	31 [19, 47]	
≥6	7	1126 [653, 2865]		7	36 [21, 43]	
Number of cardiologists			0.24 (0.15)			0.49 (0.004)
<5	14	277 [195, 548]		12	25 [12, 32]	
5–9	12	381 [207, 907]		12	34 [20, 43]	
≥10	10	365 [328, 760]		9	36 [31, 48]	
Years of experience, most senior cardiologist			0.05			0.56
≤10	7	215 [170, 293]		6	28 [20, 34]	
>10	28	455 [261, 684]		26	32 [21, 42]	
Number of ICU nurses			0.75 (<0.001)			0.15 (0.39)
<15	11	195 [110, 361]		10	32 [24, 45]	
15–39	12	311 [222, 375]		11	25 [19, 42]	
≥40	14	720 [548, 1126]		13	32 [21, 38]	
ICU bed capacity			0.76 (<0.001)			0.22 (0.20)
≤6	14	183 [110, 234]		13	26 [23, 30]	
7–15	10	381 [282, 673]		9	32 [17, 41]	
≥16	13	680 [529, 1126]		12	37 [26, 46]	
Distance travelled for surgery (percent of patients)						
> 200 km	28		0.04 (0.83)	26		0.02 (0.94)

Location in an upper-middle income country (p = 0.04), OR availability (p = 0.02), and number of cardiologists (p = 0.004) were associated with performing a higher percentage of complex cases (Table 2).

Number of surgeons (p = 0.025), anesthesiologists (p = 0.049), perfusionists (p = 0.025), cardiologists (p = 0.019), ICU nurses (p = 0.09), as well as ICU bed capacity (p = 0.01) and gross national income (GNI) per Capita (p = 0.03) were associated with having performed a higher proportion of estimated new case burden (Table 3).

**Table 3:** Association of key infrastructure to proportion of estimated new case burden performed.

	Percentage of Estimated Needed Case Volume		

	Number of Sites	Median [IQR] proportion of estimated case volume fulfilled	P-value/or Spearman r (P-value)

Operating room availability			0.11
CHD dedicated, or shared but with ≥3 ORs	26	1.02 [0.29 1.27]	
OR Shared and 1 or 2 ORs	11	0.60 [0.16, 0.79]	
Number of surgeons			0.37 (0.025)
1–2	8	0.61 [0.19, 1.03]	
3–4	15	0.59 [0.16, 1.12]	
≥5	14	1.17 [0.59, 1.46]	
Number of OR nurses			0.04 (0.83)
<4	10	0.96 [0.19, 1.27]	
4–8	15	0.59 [0.09, 0.93]	
≥9	11	0.66 [0.25, 1.97]	
Number of cardiac anaesthesiologists			0.33 (0.049)
<4	16	0.63 [0.14, 1.13]	
4–6	12	0.53 [0.34, 1.03]	
≥7	9	1.24 [0.65, 2.16]	
Number of perfusionists			0.37 (0.025)
<4	16	0.60 [0.32, 0.86]	
4–5	14	0.74 [0.09, 1.28]	
≥6	7	1.24 [0.40, 2.75]	
Number of cardiologists			0.39 (0.019)
<5	14	0.62 [0.09, 1.21]	
5–9	12	0.53 [0.24, 1.02]	
≥10	10	1.19 [0.66, 1.97]	
Number of ICU nurses			0.42 (0.009)
<15	11	0.44 [0.14, 1.11]	
15–39	12	0.63 [0.14, 1.02]	
≥40	14	1.22 [0.47, 1.28]	
ICU bed capacity			0.52 (0.001)
≤6	14	0.46 [0.16, 0.66]	
7–15	10	0.86 [0.14, 1.21]	
≥16	13	1.24 [0.63, 1.97]	
GNI per Capita	37		0.36 (0.030)

## Discussion

Healthcare advances now allow the correction or palliation of CHD, resulting in improved length and quality of life [4,5,6]. The timely diagnosis, appropriate treatment and long-term care for CHD patients is multi-disciplinary and depends on the availability of appropriately structured systems with adequate infrastructure [25,26]. There is however a large disparity in healthcare resources and access to cardiac surgery across the globe [11,12,13]. It has been estimated that 90% of the world’s children born with CHD receive suboptimal care or have no access to care [11,12].

We here present a cross-sectional study of the resource availability for CHD care at the institution/program level in 17 countries. While available studies to date have focused on specific countries, regions, or otherwise on one aspect of CHD care [18,20,27,28], we took an across the board global approach. Not surprisingly, there was an overall deficiency in the infrastructure for the care of CHD patients among the surveyed institutions, and inadequate access to care in the population areas they serve. Overall, operating room availability, intensive care capacity and availability of key staff were significant drivers of surgical case volume, case complexity mix and access to care.

The Global Alliance for Rheumatic and Congenital Hearts, a network of parent and patient advocacy groups, have issued a Declaration of Rights of Individuals Affected by Childhood-Onset Heart Disease calling for access to medical and surgical treatment for congenital and rheumatic heart disease as a basic human right (https://global-arch.org/advocacy/declaration-of-rights/). The declaration calls on governments, authorities and service providers to assume and fulfil a number of actions to help realize these rights.

Some high-income countries have established minimum requirements for staffing and infrastructure among pediatric heart surgery units [15,16,17], while others have successfully implemented regionalization as a way to promote quality assurance [29]. The WHO manual for surgical care at the district hospital offers guidelines about the necessary infrastructure for general surgery, traumatology obstetrics and traumatology. In addition, the Global Initiative for Children’s Surgery (GICS) has developed guidelines for the optimal workforce and equipment resources by subspecialty of children’s surgery at each level hospital in the health care system [30,31,32]. However, these do not offer the necessary detail regarding complex multispecialty services such as CHD patient-care. The GICS Cardiac Surgery working group is in the process of formulating such guidelines, an exercise which the granularity of detail in this report may help inform.

As countries and regions transform from low- to high-income, there is a corresponding transition from rheumatic to degenerative heart disease, coupled with an overall increase in the requirement for cardiac surgery [13]. The incidence of congenital heart disease is however uniform, imposing a greater burden on those areas with higher birth rates [2]. Moreover the distribution of health facilities in middle-income countries varies widely across location, including rural compared to urban areas [13]. Given the magnitude of the current gap in access to CHD-care in middle-income countries, a modest goal would be for existing programs to be able to care for all babies newly born with moderate or severe CHD in their coverage region or country, and to cover ongoing care for other CHD patients.

This goal could conceivably be achieved by some degree of centralization or regionalization to create well-resourced larger programs for specific regions or countries, as long as smooth patient identification, transfer and ongoing care were feasible. To ensure quality and reduce the likelihood of complications, such programs would need to have an adequate volume of CHD cases to support the maintenance of core capacities and routines [33]. The experience from Shanghai suggests that long-term investments by local governments combined with organizational collaboration with other experienced programs is a good model for capacity building over the long-term [34]. Inclusion of pediatric cardiac services in universal health coverage, public-private partnerships, prioritization of staff recruitment and collaboration in workforce training and quality initiatives [22] would further shore up and sustain CHD-care capacity.

### Study limitations

In interpreting the data presented here, it should be noted that all the respondents to the survey were from middle-income countries and none from low-income countries. No IQIC participants from low-income programs volunteered to complete the survey. Moreover, while the IQIC member institutions offered a practical study group with annually audited case data and an existing collaboration in quality improvement, they do not represent all institutions providing CHD-care everywhere. Institutions choosing to participate in the IQIC tend to be a self-selected group of high-level tertiary hospitals, which may have even more resources than other regional programs.

Another limitation is that survey responses were taken at face value and no independent verification was performed of infrastructure availability. Furthermore, in estimating new annual CHD case burden, an assumption that the healthcare facilities caring for CHD patients within a respective country or region shared the surgical burden equally may be different from the actual distribution of caseloads. Although 84% of programs also care for RHD patients, we made no attempt to estimate the number of RHD operations required because RHD burden varies greatly among middle-income countries.

## Conclusions

There exists a deficit in the structural capacity among institutions that care for CHD patients in middle-income countries. The deficit in infrastructure that requires longterm investments, specifically operating room capacity, intensive care capacity and trained staff, demonstrably limit the case volumes, complexity mix and access to care. Governments and other stakeholders should prioritize these long-range investments to match the expected number of infants born with CHD or living with CHD in specific regions, as a starting point towards achieving equitable CHD care across the globe.

## Data Accessibility Statement

De-identified aggregate infrastructure availability data may be shared for scientific purposes that fall within the framework of the IQIC data use agreements. Please email: kathy.jenkins@childrens.harvard.edu.

## Additional Files

The additional files for this article can be found as follows:

10.5334/gh.968.s1 Supplementary File 1. Supplementary tables 1 to 8.

10.5334/gh.968.s2 Supplementary File 2. CHD-Care Survey Tool.
