# Supplementary material for: Infrastructure Availability for the Care of Congenital Heart Disease Patients and Its Influence on Case Volume, Complexity and Access Among Healthcare Institutions in 17 Middle-Income Countries
Source: Glob Heart. 2021 Oct 21;16(1):75. doi: 10.5334/gh.968 (PMC8533658; doi:10.5334/gh.968)
Supplement: Supplementary File 1. — Supplementary tables 1 to 8. [file gh-16-1-968-s1.pdf]

Supplementary Table 1: The participating institutions.

Health Care Institution	Location	
Hospital de Niños	Cordoba	Argentina
Hospital Garrahan	Buenos Aires	Argentina
Dr. Carlos Alberto Studart Gomes Hospital (Hospital de Messejana)	Fortaleza	Brazil
Hospital da Criança e Maternidade (Hospital de Base)	Sao Jose do Rio Preto	Brazil
Hospital do Coracao (HCor)	Sao Paulo	Brazil
Instituto do Coracao do Hospital das Clinicas de Universidade de Sao Paulo (InCor)	Sao Paulo	Brazil
National Heart Hospital	Sofia	Bulgaria
First Hospital of Lanzhou University	Lanzhou	China
Shanghai Children's Medical Center	Shanghai	China
West China Hospital, Sichuan University	Chengdu	China
Fundación Cardioinfantil de Bogota	Bogota	Colombia
Fundacion Valle del Lili	Cali	Colombia
Hospital Nacional de Niños	San Jose	Costa Rica
JoAnn McGowan Pediatric Cardiac Surgery Center	Tbilisi	Georgia
Unidad de Cirugia Cardiovascular de Guatemala (UNICAR)	Guatemala City	Guatemala
Amrita Institute of Medical Sciences	Kochi	India
G. Kuppuswamy Naidu Memorial Hospital	Coimbatore	India
Kokilaben Dhirubhai Ambani Hospital & Medical Research Center	Mumbai	India
Narayana Hrudayalaya Institute of Cardiac Sciences		India
Public Health and Welfare Society Hospital	Chennai	India
Rabindranath Tagore International Institute of Cardiac Sciences (RTIICS)	Kolkata	India
Ramesh Hospitals	Vijayawada	India
Star Hospital	Hyderabad	India
Institut Jantung Negara	Kuala Lumpur	Malaysia
Instituto Nacional de Pediatria	Mexico City	Mexico
The American British Cowdray Medical Center, I.A.P.	Mexico City	Mexico
Aga Khan University Hospital	Karachi	Pakistan
Armed Forces Institute of Cardiology (AFIC), National Institute of Heart Disease	Rawalpindi	Pakistan
National Institute of Cardiovascular Diseases (NICVD)	Karachi	Pakistan
Instituto Nacional del Corazon (INCOR)	Lima	Peru
Federal State Budgetary Institution “Research Institute for Complex Problems of Cardiovascular Diseases”, Siberian Branch of the Russian Academy of Medical Science	Kemerovo	Russia
Nizhny Novgorod Cardiac Surgical Center	Nizhny Novgorod	Russia
Mother and Child Health Institute	Belgrade	Serbia
Institute of General & Urgent Surgery, Academy of Medical Science	Kharkiv	Ukraine
Nhi Dong 1 (Children's Hospital #1)	Ho Chi Minh City	Vietnam
Tam Duc Heart Hospital	Ho Chi Minh City	Vietnam
Vietnam National Children's Hospital	Hanoi	Vietnam

Supplementary Table 2: Availability of operating room infrastructure and equipment stratified by program location and program site

Infrastructure Variable		All Programs (N=37)	Program Location: Americas (N=13)	Program Location: Asia (N=18)	Program Location: Eastern Europe (N=6)	Program Size†: Small-Med (N=13)	Program Size†: Large (N=9)	Program Size†: Very Large (N=15)
**Operating Room**								
Operating Room/s (OR) Median (Range)		2 (1-16)	2 (1-7)	2 (1-16)	1 (1-2)	1 (1-3)	3 (1-7)	2 (2-16)
OR dedicated	CHD dedicated*	16 (43%)	5	8	3	5	2	9
	Specialist share*	17 (46%)	5	9	3	7	5	5
	General share*	4 (11%)	3	1	0	1	2	1
Unidirectional Airflow Installed and Functioning	All OR	31 (83%)	12	16	3	10	8	13
	Some OR	1 (3%)	0	1	0	0	0	1
	None	5 (14%)	1	1	3	3	1	1
Appropriate Temperature Control is installed	Heat and Cool	28 (76%)	11	12	5	10	6	12
	Only cool	9 (24%)	2	6	1	3	3	3
Suction System is available in OR	All	36 (97%)	13	17	6	13	9	14
	Some	1 (3%)	0	1	0	0	0	1
Adequate overhead lighting		37 (100%)						
Extra forehead mounted lighting available		31 (84%)	12	15	4	10	9	12
Defibrillator and Internal Paddles Available	All OR	36 (97%)	13	17	6	13	9	14
	Some OR	1 (3%)	0	1	0	0	0	1
Rapid Fibrillator available		18 (51%)	8	8	2	4	6	8
Surgical telescopes (Loops) available		37 (100%)						
Photographic &Recording equipment is available		16 (43%)	6	8	2	6	2	8
**Perfusion Equipment**								
CPB machine Median (Range)		2 (2-4)	2 (2-6)	3 (3-5)	2 (2-2)	2 (2-3)	3 (2-5)	3 (2-6)
CPB machine dedicated	CHD dedicated*	19 (51%)	5	10	4	7	4	8
	Specialist share*	17 (46%)	8	7	2	6	5	6
	General share*	1 (3%)	0	1	0	0	0	1
Sealed Bypass packs available	Always	34 (92%)	12	17	5	12	8	14
	Sometimes	2 (5%)	1	0	1	1	1	0
	Never	1 (3%)	0	1	0	0	0	1
Sealed Cardioplegia Tubing available	Always	32 (86%)	12	15	5	11	8	13
	Sometimes	2(5%)	1	0	1	1	1	0
	Never	3 (8%)	0	3	0	1	0	2
Arterial and Venous Cannula available	Always	33 (89%)	11	17	5	11	8	14
	Sometimes	3(8%)	2	0	1	2	1	0
	Never	1(3%)	0	1	0	0	0	1
**Instrument Sterilization and Disposables**								
Sterilization methods available	High/Low Temp **	29 (78%)	10	15	4	9	8	12
	Only High Temp **	4 (11%)	2	1	1	2	0	2
	No Steam **	4 (11%)	1	2	1	2	1	1
OR Instrument replacement practice	Yearly	4 (11%)	2	1	1	1	1	2
	1-3 Years	6 (16%)	5	1	0	1	2	3
	Need-by basis	27(73%)	6	16	5	11	6	10
Sourcing of disposable materials for operations	Centrally	33 (89%)						
	Donations	3 (8%)						
	Patients	1 (3)						
Reuse of single use items	Never	15 (40%)	5	7	3	6	5	4
	Rarely	5 (13%)	1	2	2	2	1	2
	Sometimes	7 (14%)	2	5	0	2	1	4

	Often	7 (14%)	3	3	1	2	1	4
	Always	3 (9%)	2	1	0	1	1	1
Programs that re-use Arterial or Venous Cannula***		14 (37%)						
Reuse of expired items	Never	32 (86%)	11	17	4	11	7	14
	Rarely	2 (5%)	0	1	1	0	1	1
	Sometimes	0 (0%)	0	0	0	0	0	0
	Often	2 (6%)	2	0	0	1	1	0
	Always	1 (3%)	0	0	1	1	0	0

*"CHD dedicated" indicates infrastructure dedicated to the congenital heart disease team. *"Specialist share" indicates infrastructure shared by the CHD program with closely related specialists like adult cardiac surgeons, vascular surgery or pediatric surgery. *"General Share" indicates. Infrastructure that is shared generally with other teams working in the hospital.

"High/Low Temp" indicates availability of Steam, Flash or Dry high temperature sterilization systems and at least one low temperature chemical sterilization system like hydrogen peroxide or ethanol. ** "Only High Temp" indicates those programs with only high-temperature sterilization systems. "No Steam" Indicates programs without access to steam sterilization.

All percentages are rounded off to the nearest full number.

CPB=cardiopulmonary bypass; Temp = Temperature.

† Program size; Small to Medium =>250 total cases in the last year; Large=250-500 cases; Very large=More than 500 cases.

Supplementary Table 3: New categories formed by combining responses

<table>
<tr><th>New Category</th><th>Component Categories</th><th>How Responses were combined</th></tr>
<tr><td>Synthetic Vascular Conduits</td><td>Goretex/PFTE vascular conduits, Dacron vascular conduits</td><td rowspan="16">The responses were reclassified as follows.
ALWAYS=Always is checked in In ANY ONE of the component questions.
SOMETIMES =Sometimes, (Always is NOT CHECKED in ANY of the component questions AND Sometimes IS CHECKED in at least One of the component questions).
NEVER= Never, (Always and Sometimes ARE NOT CHECKED in any of the component questions AND never is checked in at least one of the component questions)</td></tr>
<tr><td>Synthetic Vascular Patches</td><td>Goretex/PFTE vascular patch, Dacron vascular patch</td></tr>
<tr><td>Treated Pericardium Tissue Patches</td><td>PhotoFix bovine pericardium patches, Gultaraldehyde-fixed bovine pericardium patches</td></tr>
<tr><td>Mechanical Mitral Valves</td><td>Bileaflet mechanical aortic valves, Tilting disk mechanical aortic valve</td></tr>
<tr><td>Biological Mitral Valves</td><td>Bovine mitral valves, Porcine mitral valves</td></tr>
<tr><td>Mechanical Aortic Valves</td><td>Bileaflet mechanical aortic valves, Tilting disk disk mechanical aortic valves</td></tr>
<tr><td>Biological Aortic Valves</td><td>Bovine aortic valves, Porcine aortic valves</td></tr>
<tr><td>Mechanical Pulmonary valves</td><td>Bileaflet mechanical pulmonary valves, Tilting disk mechanical pulmonary valves</td></tr>
<tr><td>Biological Pulmonary valves</td><td>Bovine pulmonary valves, Porcine biological valves</td></tr>
<tr><td>Mechanical Tricuspid Valves</td><td>Bileaflet mechanical tricuspid valves, Tilting disk mechanical tricuspid valves</td></tr>
<tr><td>Biological Tricuspid Valves</td><td>Bovine tricuspid valves, Porcine tricuspid valves</td></tr>
<tr><td>Propofol OR Sodium thiopental</td><td>Propofol,Sodium thiopental</td></tr>
<tr><td>Volatile Anesthetics 1</td><td>Halothane, Isoflurane</td></tr>
<tr><td>Volatile Anesthetics 2</td><td>Sevoflurane, Desflurane</td></tr>
<tr><td>Vasopressor drugs</td><td>Dopamine, Dobutamine, Norepinephrine, Epinephrine, Epinehrine,Vasopressin</td></tr>
<tr><td>Morphine OR Fentanyl</td><td>Morphine, Fentanyl</td></tr>
<tr><td>Transesophageal Echocardiography or Epicardial Echocardiography</td><td>Transesophageal Echocardiography, Epicardial Echocardiography</td><td>YES in either Response,
NO in both responses</td></tr>
</table>

Supplementary Table 4: Estimated annual cases with moderate or severe CHD, as well as estimated need CHD case need at the participating sites.

Country (or Region)	2016 Population of Country (or Region)	2016 Crude Birth Rate of Country or Region (per 1000 population)	Estimated Annual Live Births in 2016	Estimated Babies Born with Moderate or Severe Forms of CHD (0,6% of live Births)	Estimated number of Institutions in the Country (or Region) Providing regular CHD Services	Estimated new case-burden of CHD Patients at Institution level in 2016.
Argentina	43,590,368	17.38	757,601	4,546	10	455
Brazil	203,627,284	14.30	2,913,906	17,483	81	216
Bulgaria	7,127,822	9.1	64,863	389	1	389
China (Gansu)	26,100,000	12.18	317,898	1,907	3	636
China (Sichuan)	82,620,000	10.48	865,857	5,195	11	472
China (Shanghai)	24,200,000	9	217,800	1,307	4	327
Colombia	48,171,392	15.31	737,504	4,425	20	221
Costa Rica	4,899,345	14.51	71,089	427	1	427
Georgia	3,727,505	13.93	51,924	312	1	312
Guatemala	16,583,060	25.27	419,054	2,514	1	2,514
India (Andra Pradesh)	53,060,000	19.01	1,008,671	6,052	3	2,017
India (Karnataka)	66,800,000	19.01	1,269,868	7,619	4	1,905
India (Kerala)	34,279,669	14.48	496,292	2,978	6	496
India (Maharashtra)	118,652,000	15.9	1,886,567	1,1319	5	2,264
India (Tamil Nadu)	79,788,000	19.01	1,516,770	9,100	20	455
India (Telangana)	38,600,000	17.8	687,080	4,122	6	687
India (West Bengal)	95,416,000	19.01	1813858	10883	4	2,721
Malaysia	30,684,804	16.96	520,414	3,122	3	1,041
Mexico	123,333,376	18.245	2,250,212	13,501	22	614
Pakistan	203,627,284	28.88	5,880,756	35,285	5	7,057
Peru	30,926,032	18,32	566,565	3,399	3	1,133
Russian Federation	144,342,396	12.9	1,862,017	11,172	78	143
Serbia	7,058,322	9.2	64,937	390	3	130
Ukraine	45004645	10.3	463548	2781	8	348
Vietnam	93638724	17.15	1605904	9635	19	507

Number of facilities providing CHD services in the country or region was evaluated according to information from respondents, crosschecked with available literature, and supplemented by internet research. Facilities were counted if they routinely provide CHD and performed at least an estimated 30 CHD cases per year.

Supplementary Table 5: Anesthesia and post-operative care infrastructure stratified by location and program size

Infrastructure Variable		All Programs (N=37)	Program Location: Americas (N=13)	Asia (N=18)	Eastern Europe (N=6)	Program Size†: Small-Med (N=13)	Large (N=9)	Very Large (N=15)
Anaesthesia machines	CHD dedicated*	14 (38%)	3	8	3	5	2	7
	Specialist share*	18 (49%)	6	9	3	7	5	6
	General share*	5 (14%)	4	1	0	1	2	2
Neurological monitoring during anaesthesia (N=36)	BSI	8 (22%)	3	5	0	0	5	3
	Near Infrared	13 (35%)	8	3	2	6	2	5
	None	15 (41%)	2	9	4	7	2	6
Intra-op echocardiography	TEE (N=36)	31 (86%)	10	16	5	11	7	13
	TTE	33 (89%)	11	16	6	13	6	14
	Both	29 (78%)	10	14	5	11	6	12
	Neither	2 (5%)	2	0	0	0	2	0
Infrastructure for cooling and warming the patient	Counter current exchanger	34 (92%)	13	15	6	12	8	14
	Ice bucket slush	3 (8%)	0	3	0	1	1	1
Core-body Temperature Monitoring	Oesophageal or Bladder	32 (87%)	13	14	5	12	6	14
	Did NOT check above AND checked Rectal, Tympanic or Nasal or Skin	5 (13%)	0	4	1	1	3	1
Co-oximeter available		32 (86%)	10	17	5	10	8	14
Adequate cartridges available for co-oximeter		29 (78%)	10	16	3	8	8	13
Perform regular ACT		35 (95%)	12	17	6	13	8	14
Monitor end-tidal C02	Always	33 (89%)	13	14	6	13	8	12
	Sometimes	4 (11%)	0	4	0	0	1	3
	Never	0 (0%)	0	0	0	0	0	0
Monitor CVP	Always	35 (95%)	11	18	6	13	7	15
	Sometimes	2 (5%)	2	0	0	0	2	0
	Never	0 (0%)	0	0	0	0	0	0
PH management strategy (N=36)	Alphastat	15 (42%)	3	8	4	8	3	4
	PHstat	21 (58%)	10	9	2	5	6	10
Modified Ultrafiltration		32 (86%)	11	16	5	10	8	14
Blood bank on Site		29 (81%)	11	14	4	8	8	13
**Post-operative Care**								
ICU care for paediatric CHD patients	CHD dedicated*	22 (60%)	11	7	4	8	5	9
	Cardiac Shared*	11 (30%)	1	8	2	3	3	5
	Peds Shared*	4 (10%)	1	3	0	2	1	1
ICU care for adult CHD patients	With ped. CHD	17 (46%)	6	10	1	4	4	9
	Adult cardiac	15 (41%)	6	5	4	7	4	4
	Other	5 (13%)	1	3	1	2	1	2
ICU bed capacity		9 (2-80)	9 (3-30)	13(2-80)	6 (4-9)	6 (2-16)	9 (4-24)	20 (6-80)
Typical bed capacity of ICU		3 (1-45)	3(1-13)	4(4-25)	2(1-5)	2(1-7)	2(2-8)	5(2-45)
Separate NICU for post-ops		14 (38%)	6	7	1	7	2	5
Ventilators for Neonates		36 (97%)	13	17	6	13	9	14
High Frequency oscillators		20 (54%)	9	8	3	7	4	9
Incubators		29 (78%)	9	14	6	10	6	13
Warming Tables		30 (83%)	11	13	6	10	8	12
Dedicated code carts (N=36)		33 (92%)	11	17	5	12	7	14
Emergency medication in code cart		33(89%)	11	17	5	12	7	14
Pediatric formulations in emergency meds	All	18 (55%)	5	10	3	6	4	8
	Some	8 (24%)	2	4	2	5	1	2
	None	7 (21%)	4	3	0	1	2	4

Sterile emergency instruments		30 (81%)	8	16	6	10	6	14
Cath lab on site or nearby	Onsite	36 (97%)	12	18	6	12	9	15
	Nearby	1 (3%)	1	0	0	1	0	0
Echo within an hour in case of emergency		34 (92%)	11	18	5	11	8	15
		3 (8%)	2	0	1	2	1	0
Echo under sedation for neonates		36 (97%)	13	17	6	13	8	15
ECMO program		20 (54%)	7	10	3	3	5	12
CT		30 (81%)	10	16	4	10	6	14
MRI		20 (54%)	7	11	2	4	3	13
Nuclear Imaging		17 (46%)	6	10	1	3	4	10
Have an intermediate care ward		18 (50%)	3	14	1	4	4	10
Intermediate care ward capacity		7 (3-36)	6 (5-23)	7(3-36)	8(8-8)	4(3-8)	6(5-11)	10(4-36)
Intermediate care ward typical occupancy (N=17)		3(1-27)	2(2-6)	3 (1-27)	2 (2-2)	2(1-2)	3(1-6)	3(2-27)
General ward capacity		18 (4-45)	11(4-30)	25(20-80)	18(8-30)	9(4-30)	18(10-20)	28(20-85)

Supplementary Table 6: Availability of critical staff stratified by program location and size

Overall Staff Numbers	All Programs (N=37)	Americas (N=13)	Asia (N=18)	Europe (N=6)	Small-Med (<250)	Large (250-500)	Very Large (>500)
Surgeons	3 (1 – 20)	3 (2 – 6)	5 (1 – 20)	3 (2 – 14)	3 (1 – 5)	5 (1 – 7)	5 (2 – 20)
OR Nurses	5 (0 – 30)	5 (0 – 15)	6 (3 – 30)	4 (2 – 5)	4 (1 – 6)	10 (0 – 15)	6 (2 – 30)
Perfusionists							
Anesthesiologists	4 (2 – 35)	5 (2 – 20)	5 (2 – 35)	3 (2 – 7)	3 (2 – 8)	3 (2 – 11)	5 (2 – 35)
Non physician anaesthesology provider	0 (0 – 17)	0 (0 – 15)	3 (0 – 17)	0 (0 – 2)	0 (0 – 5)	0 (0 – 10)	2 (0 – 17)
Pediatric Intensive care physicains	6 (0 – 20)	7 (3 – 20)	4 (0 – 13)	6 (2 – 8)	6 (0 – 8)	5 (3 – 13)	7 (1 – 20)
Intensive care nurses	28 (5 – 194)	19 (8 – 70)	40 (5 – 194)	14 (7 – 33)	11 (5 – 46)	28 (8 – 52)	50 (12 – 194)
Nurses aides/nursing technicians ICU	3 (0 – 220)	18 (0 – 88)	2 (0 – 220)	0 (0 – 7)	0 (0 – 31)	10 (0 – 88)	3 (0 – 220)
Respiratory therapists	3 (0 – 30)	8 (0 – 30)	3 (0 – 10)	0 (0 – 4)	2 (0 – 10)	1 (0 – 17)	4 (0 – 30)
Cardiologists	6 (0 – 30)	8 (2 – 30)	5 (0 – 20)	6 (1 – 15)	4 (0 – 15)	10 (4 – 20)	5 (2 – 30)
Nurses on step-down or regular wards	9 (2 – 80)	9 (2 – 80)	9 (2 – 65)	11 (4 – 18)	8 (3 – 30)	8 (2 – 19)	33 (4 – 80)
Regular visiting team	2 (1 – 11)	2 (2 – 3)	2 (1 – 11)	2 (1 – 4)	2 (1 – 10)	6 (2 – 11)	2 (1 – 2)

Supplementary Table 7: Structural and maintenance infrastructure stratified by program location and size

Infrastructure Variable		All Programs (N=37)	Program Location<br>Americas (N=13)	 Asia (N=18)	 Eastern Europe (N=6)	Program Size†<br>Small-Med (N=13)	 Large (N=9)	 Very Large (N=15)
Main Power Supply	National Grid	34 (92%)	11	17	6	13	8	13
	Generator	3 (8%)	2	1	0	0	1	2
Backup power supply available (N = 36)		34 (94%)	13	17	4	11	9	14
Backup power Covers entire hospital (N=34)		28 (82%)	9	15	4	9	7	12
		6 (18%)	4	2	0	2	2	2
Power surge protectors installed		33 (92%)	10	18	5	11	7	15
Back up water supply available		23 (62%)	7	14	2	7	6	10
Dedicated Cleaning of Hospital Spaces	Environmental Service	18 (49%)	6	12	0	4	3	11
	Regular staff	12 (32%)	1	5	6	6	4	2
	Outside Company	7(19%)	6	1	0	3	2	2
Medical Disposal System	Nationwide	13 (35%)	4	8	1	5	2	6
	Contractor	19(51%)	8	8	3	5	6	8
	Onsite	5(14%)	1	2	2	3	1	1
Medical Records System	Electronic	5 (13%)	4	1	0	1	1	3
	Mixed	24 (65%)	6	13	5	9	5	10
	Paper	8 (22%)	3	4	1	3	3	2
System for hospital order communication	Electronic	15 (41%)	7	5	3	5	2	8
	Mixed	1 (2%)	0	1	0	0	0	1
	Paper	21 (57%)	6	12	3	8	7	6
Biomedical Engineering for equipment Maintenance	In Hospital	30 (81%)	11	17	2	9	7	14
	External	5(14%)	1	1	3	3	1	1
	None	2(5%)	1	0	1	1	1	0
Cardiovascular equipment maintained to manufacturer specifications	Always	22 (61%)	8	11	3	8	5	9
	Often	7 (19%)	0	5	2	1	2	4
	Sometimes	3 (8%)	2	1	0	1	1	1
	Rarely	4 (11%)	3	0	1	3	1	0
	Never	0 (0%)	0	0	0	0	0	0
Manufacturer warranty of ICU equipment		32 (86%)	10	18	4	7	6	15
Manufacturer warranty of OR equipment		29 (83%)	10	15	4	10	7	12

Supplementary Table 8: Items commonly resterilized and reused.

**Commonly reused single use**	Number of Institutions that mentioned item.
Arterial or venous cannula	14
Vascular patch materials	6
Vascular conduits	4
Suction tubes, vents and sumps	4
Cardioplegia cannulas	3
Torniquets	1
Ventilator circuits	1
CPB circuits	1
Almost all except syringes	1
Guidewire for central monitoring cannulae	1
Vessel loops	1
Connectors	1
Cautery blades	1
Suture protector material for clamp tips	1
**Commonly re-used items after recommended use-by date**	**Number of Institutions.**
Tracheostomy cannulas	1
Annuloplasty rings	1
Everything	1
Sutures	1
Arterial or Venous cannulae	1
