# Supplementary material for: Infrastructure Availability for the Care of Congenital Heart Disease Patients and Its Influence on Case Volume, Complexity and Access Among Healthcare Institutions in 17 Middle-Income Countries
Source: Glob Heart. 2021 Oct 21;16(1):75. doi: 10.5334/gh.968 (PMC8533658; doi:10.5334/gh.968)
Supplement: Supplementary File 2. — CHD-Care Survey Tool. [file gh-16-1-968-s2.pdf]

Section 1. Operating Room

SECTION 1: OPERATING ROOM

Operating Room Infrastructure

1. How many operating room(s) do you have for the conduct of pediatric cardiac and adult congenital cardiac operations?

____

Please choose the option that best describes the resource allocation of this/those operating room(s):

◯ The operating room(s) is/are dedicated for pediatric cardiac and adult congenital cardiac operations.
◯ The operating room(s) is/are shared for all pediatric and adult cardiac operations (congenital and acquired).
◯ The operating room(s) is/are used for cardiac and non-cardiac operations but dedicated for only pediatric patients .
◯ The operating room(s) is/are used for all types of surgery and all ages of patients.
◯ Other

If other, please describe:

2. How is ambient room temperature regulated in the above operating room(s)?

◯ There is a functioning system to both heat and cool the operating room(s) to the desired ambient temperature.
◯ There is a functioning system for heating only but no functioning cooling system.
◯ There is a functioning system for cooling only but no functioning heating system.
◯ There is no specific temperature regulation in the operating room(s).
◯ Other

If other, please describe:

3. Is unidirectional air ventilation installed and functioning in the above operating room(s)?

◯ Yes, all of our operating rooms have unidirectional air ventilation installed and functioning.
◯ Some of our operating rooms have unidirectional air ventilation installed and functioning.
◯ No, our operating room(s) does not have functioning unidirectional air ventilation.

4. Is there vacuum (negative) suction, either portable or central (wall suction), available in the above operating room(s)?

◯ Yes, suctioning is available in all operating rooms.
◯ Suctioning is available in some operating rooms.
◯ No, suctioning is not available in the operating room(s).

5. Is there adequate lighting in the above operating room(s)?

◯ Yes
◯ No

If no, please describe:

6. Do you have any limitations in your operating room(s) infrastructure that restrict the number and complexity of pediatric cardiac and adult congenital cardiac operations that your team can perform?

◯ Yes
◯ No

If yes, please describe:

Surgical Instruments and Equipment

7. What system(s) do you use for the sterilization of surgical instruments? Please check all that apply.

☐ Steam sterilization
☐ Flash steam sterilization
☐ Ethylene oxide sterilization
☐ Hydrogen peroxide gas sterilization
☐ Dry heat sterilization
☐ Other

If other, please describe:

8. How often are new surgical instruments acquired and old ones replaced?

◯ Every year
◯ Every1-3 years
◯ On a need basis
◯ Other

If other, please specify

9. Please describe what resources you have available for the cleaning of surgical equipment (e.g. Bypass machine).

10. Please describe what resources you have available for the cleaning of the operating room(s) space.

11. Please describe what resources you have available for the cleaning of surgical scrubs and linens.

12. How often do you re-sterilize and re-use items that are typically for single-use (i.e., as indicated by the manufacturer)?

◯ Never ◯ Rarely ◯ Sometimes ◯ Often ◯ Always

Please indicate which single-use items you re-sterilize and re-use:

13. How often do you use surgical devices beyond their manufacturer recommended expiration dates?

◯ Never ◯ Rarely ◯ Sometimes ◯ Often ◯ Always

Please indicate which surgical devices you use beyond their manufacturer recommended expiration dates:

14. Which of the following equipment is available to surgeons for the conduct of pediatric cardiac surgery and adult congenital cardiac surgery? Check all that apply.

☐ Surgical telescopes (loops)
☐ Extra forehead mounted lighting
☐ Photographic/video recording equipment

15. Do you have a defibrillator and internal defibrillation paddles available in the operating room(s)?

◯ Yes, both a defibrillator and internal paddles are available in all operating rooms.
◯ Both a defibrillator and internal paddles are available in some operating rooms.
◯ We have a defibrillator but no internal paddles available.
◯ No, we have neither a defribrillator nor internal paddles available in the operating room(s).
◯ Other

If other, please describe:

Do you have access to a cardiac rapid fibrillator device for the conduct of whole or parts of some cardiac procedures without arresting the heart?

◯ Yes
◯ No

16. Do you have any limitations related to operating room(s) instruments and/or equipment that restrict the number and complexity of pediatric cardiac and adult congenital cardiac operations that your team can perform?

◯ Yes
◯ No

If yes, please describe:

Prosthetic Materials and Devices

17. How are prosthetic materials and devices primarily sourced for your cardiac surgery cases?

◯ Prosthetic materials and devices are centrally purchased by the hospital/medical center for use in all cases as deemed appropriate by the surgeon.
◯ Prosthetic materials and devices are used as available from donations.
◯ Prosthetic materials and devices are bought individually by the patients themselves depending on cost and availability.
◯ Other

If other, please describe:

18. Please select the option that best describes the availability of prosthetic materials, sutures, and devices to your team for the conduct of cardiac surgery cases.

Prosthetic Conduits and Patches

	Always	Sometimes	Never
Homograft vascular conduits	◯	◯	◯
Bovine vascular conduits (e.g., Contegra)	◯	◯	◯
Self-made autologous pericardial vascular conduits	◯	◯	◯
Goretex/PFTE vascular conduits	◯	◯	◯
Dacron vascular conduits	◯	◯	◯
PhotoFix bovine pericardium patches	◯	◯	◯

Gultaraldehyde-fixed bovine pericardium patches	◯	◯	◯
Goretex/PFTE patches	◯	◯	◯

Prosthetic Valves

	Always	Sometimes	Never
Bileaflet mechanical mitral valves	◯	◯	◯
Tilting disk mitral valves	◯	◯	◯
Bovine mitral valves	◯	◯	◯
Porcine mitral valves	◯	◯	◯
Mitral valve annuloplasty rings	◯	◯	◯
Bileaflet mechanical aortic valves	◯	◯	◯
Tilting disk mechanical aortic valves	◯	◯	◯
Bovine aortic valves	◯	◯	◯
Porcine aortic valves	◯	◯	◯
Bileaflet mechanical pulmonary valves	◯	◯	◯
Tilting disk mechanical pulmonary valves	◯	◯	◯
Bovine biological valves	◯	◯	◯
Porcine biological valves	◯	◯	◯
Bileaflet mechanical tricuspid valves	◯	◯	◯
Tilting disk mechanical tricuspid valves	◯	◯	◯
Bovine tricuspid valves	◯	◯	◯
Porcine tricuspid valves	◯	◯	◯
Tricuspid valve annuloplasty rings	◯	◯	◯

Pacing Materials

	Always	Sometimes	Never
Temporary pacing wires	◯	◯	◯
Permanent pacemakers and leads	◯	◯	◯

19. Do you have any limitations related to prosthetic materials and devices that restrict the number and complexity of pediatric cardiac and adult congenital cardiac operations that your team can perform?

◯ Yes
◯ No

If yes, please describe:

Intraoperative Monitoring and Anesthesia

20. Which option best describes the anesthetic machine(s) available for the conduct of pediatric cardiac and adult congenital cardiac operations at your hospital/medical center?

◯ The anesthetic machine(s) is/are dedicated for pediatric and adult congenital cardiac operations.
◯ The anesthetic machine(s) is/are used in all types of pediatric operations.
◯ The anesthetic machine(s) is/are shared for all pediatric cardiac and adult cardiac operations.
◯ The anesthetic machine(s) is/are used for any type of operation, regardless of patient age.
◯ Other

If other, please describe:

21. Please specify the name, model, and year of manufacture of your above anesthetic machine(s) .

22. Please indicate the availability of the following essential medical and anesthetic gases for the conduct of pediatric cardiac anesthesia.

Medical Gas

	Always	Sometimes	Never
Oxygen	◯	◯	◯
Nitrous oxide	◯	◯	◯
Carbon dioxide	◯	◯	◯
Compressed room air	◯	◯	◯
Halothane	◯	◯	◯
Isoflurane	◯	◯	◯
Sevoflurane	◯	◯	◯
Desflurane	◯	◯	◯

23. Please indicate the availability of the following essential anesthetic and medical medications for the conduct of pediatric cardiac anesthesia at your medical facility.

Drug

	Always	Sometimes	Never
Ketamine	○	○	○
Propofol	○	○	○
Etomidate	○	○	○
Diazepam or Lorazepam	○	○	○
Sodium thiopental	○	○	○
Atropine	○	○	○
Succinylcholine	○	○	○
Dopamine	○	○	○
Dobutamine	○	○	○
Norepinephrine	○	○	○
Epinephrine	○	○	○
Vasopressin	○	○	○
Milrinone	○	○	○
Nitroglycerin	○	○	○
Lidocaine	○	○	○
Procainamide	○	○	○
Lasix	○	○	○
Morphine	○	○	○
Fentanyl	○	○	○
Heparin	○	○	○
Protamine	○	○	○
Magnesium Sulphate	○	○	○
Calcium gluconate or Chloride	○	○	○
Insulin	○	○	○
50% dextrose	○	○	○
5% or 25% pooled albumin	○	○	○
Prostaglandin E	○	○	○

24. Please specify how often you use the following types of cardiovascular monitoring during pediatric cardiac and adult congenital cardiac operations at your hospital/medical center.

Cardiovascular Monitoring

	Always	Sometimes	Never
Electrocardiogram (EKG)	○	○	○
Pulse oximetry	○	○	○

End tidal CO2	◯	◯	◯
Invasive blood pressure monitoring (arterial line)	◯	◯	◯
Central venous pressure	◯	◯	◯

25. What is your strategy for intraoperative neurologic monitoring?

◯ Bispectral index (BIS)
◯ Near-infrared spectroscopy
◯ No intraoperative neurologic monitoring is performed
◯ Other

If other, please describe:

26. Do you have access to intraoperative transesophageal echocardiography (TEE), if required?

◯ Yes
◯ No

27. Do you have access to intraoperative epicardial echocardiography?

◯ Yes
◯ No

28. How do you monitor a patient's temperature intraoperatively? Please check all that apply.

☐ Esophageal temperature probe
☐ Bladder temperature probe
☐ Rectal temperature probe
☐ Tympanic temperature probe
☐ Other

If other, please describe:

29. Do you have a dedicated co-oximeter for intraoperative assessment of blood gases?

◯ Yes
◯ No

If yes, do you have access to an adequate supply of cartridges for the conduct of pediatric cardiac and adult congenital cardiac surgery operations?

◯ Yes
◯ No

If no, please describe:

30. Do you have access to intraoperative bedside activated clotting time assessment?

◯ Yes
◯ No

31. What type of electrolyte assessment do you routinely conduct during pediatric cardiac and adult congenital cardiac operations?

◯ Blood sugar monitoring
◯ Basic metabolic panel monitoring
◯ We do not routinely conduct electrolyte assessment during pediatric and adult congenital cardiac operations.
◯ Other

If other, please describe:

32. Please select the option that best describes the availability of blood products used in the conduct of pediatric cardiac and adult congenital cardiac operations at your hospital/medical center.

Blood Product

	Always	Sometimes	Never
Whole blood	◯	◯	◯
Red blood cells	◯	◯	◯
Fresh frozen plasma	◯	◯	◯
Platelets	◯	◯	◯
Cryoprecipitate	◯	◯	◯
Antithrombin III	◯	◯	◯
Activated factor VII (Novoseven)	◯	◯	◯
Other	◯	◯	◯

If other, please describe:

33. Do you have a blood bank on site for the supply and monitoring of blood products?

◯ Yes, a blood bank is on site
◯ No, but a blood bank is nearby (less than 1 hour's drive)
◯ No, and the next blood bank is far away (more than 1 hour's drive)
◯ Other

If other, please describe:

34. Do you have any limitations related to cardiac intraoperative monitoring or anesthesia at your institution?

◯ Yes
◯ No

If yes, please describe:

Perfusion

35. Which option best describes the cardiopulmonary bypass machine(s) available for the conduct of pediatric and adult congenital cardiac operations at your hospital/medical center:

◯ The cardiopulmonary bypass machine(s) is/are dedicated for pediatric cardiac and adult congenital cardiac operations.
◯ The cardiopulmonary bypass machine(s) is/are used in all types of pediatric operations.
◯ The cardiopulmonary bypass machine(s) is/are shared for all pediatric cardiac and adult cardiac operations.
◯ The cardiopulmonary bypass machine(s) is/are used for any type of operation, regardless of patient age.
◯ Other

If other, please describe:

36. Do you have more than one cardiopulmonary bypass machine such that operations can continue in case one fails?

◯ Yes
◯ No

If yes, how many cardiopulmonary bypass machines do you have?

____

Please specify the name, model, and year of manufacture of your cardiopulmonary bypass machine(s).

37. Please select the option that best describes the availability of perfusion materials used in the conduct of pediatric cardiac and adult congenital cardiac operations at your hospital/medical center.

Perfusion Material

	Always	Sometimes	Never
Sealed and sterile single-use bypass packs	◯	◯	◯
Arterial and venous cannulae	◯	◯	◯
Sealed and sterile cardioplegia packs	◯	◯	◯

38. Is a cardioplegia solution available?

◯ Yes, we have access to commercially available cardioplegia solution.
◯ Yes, we have access to locally mixed cardioplegia solution.
◯ No, cardioplegia solution is not available.

39. What is your approach for cooling and warming the patient during cases?

◯ Inline warmer and cooler using coils with countercurrent flow
◯ Inline bath with either warm saline or ice slush added
◯ Other

If other, please describe:

40. What is your strategy for pH management of neonates and children during hypothermic cardiopulmonary bypass?

◯ pH-stat method
◯ Alpha-stat method
◯ Other

If other, please describe:

41. Do you utilize modified ultrafiltration (MUF) strategy in your pediatric bypass cases?

◯ Yes, we always utilize MUF in our pediatric bypass cases.
◯ Yes, we sometimes utilize MUF in our pediatric bypass cases.
◯ No, we do not utilize MUF.

42. Do you have any limitations related to cardiac perfusion at your institution that restricts the number and complexity of pediatric cardiac and adult congenital cardiac operations that your team can perform?

◯ Yes
◯ No

If yes, please describe:

Section 2. Catheterization Laboratory

SECTION 2. CATHETERIZATION LABORATORY

1. Does your hospital/medical center have a catheterization laboratory either onsite or nearby?

◯ Yes, we have catheterization laboratory onsite
◯ Yes, we have an associated catheterization laboratory nearby
◯ No, we do not have a catheterization laboratory

If no, where do your patients undergo catheterization?

◯ Our patients do not undergo catheterization
◯ Other

If other, please describe:

2. Is the catheterization laboratory dedicated to congenital cardiac catheterizations?

◯ Yes the catheterization laboratory is dedicated for congenital cardiac catheterizations.
◯ The catheterization laboratory is shared for congenital and adult acquired cardiac catheterizations.
◯ The catheterization laboratory is shared for cardiac and vascular catheterizations.
◯ Other

If other, please describe:

3. Please specify the name, model, and year of manufacture of your catheterization laboratory main fluoroscopic machine(s).

4. Is your fluoroscopy machine(s) single plane or biplane?

◯ Single plane
◯ Biplane

5. Please provide the details of the contrast material that you principally use for cases (name and manufacturer):

6. Is cardiac surgery backup available in your center for routine pediatric cardiac and adult congenital cardiac catheterization cases, if needed?

◯ Yes, cardiac surgery backup is always available
◯ Cardiac surgery backup is sometimes available
◯ No, cardiac surgery backup is not routinely available

7. Do you have an integrated procedural suite that combines the tools and equipment available in a cardiac catheterization laboratory with the sterility standards and surgical facilities of an operating room for the conduct of hybrid procedures?

◯ Yes
◯ No

If yes, how many hybrid procedures were conducted in 2015?

____

8. Do you have a functioning co-oximeter (blood gas analyzer) in the catheterization laboratory or nearby for the direct measurement of blood oxygen saturation during catheterization cases?

◯ Yes, it is dedicated to the catheterization laboratory
◯ Yes, it is shared with other clinical areas
◯ No, we do not have a functioning co-oximeter

9. Do you have a functioning pressure transducer system for the direct assessment of intravascular and intracardiac pressures during catheterization cases?

◯ Yes, and the system is dedicated to the catheterization laboratory
◯ Yes, but the system is shared with other clinical areas
◯ No, we do not have a functioning pressure transducer system

10. How are disposable supplies principally sourced for individual pediatric and adult congenital cardiac catheterizations? Check all that apply.

◯ All cardiac catheterization disposable supplies are centrally purchased by the hospital/medical center for use in all cases as deemed appropriate by the interventionalist.
◯ Cardiac catheterization disposable supplies are used as available from donations.
◯ Cardiac catheterization disposable supplies are bought individually by the patients themselves depending on cost and availability.
◯ Other

If other, please describe:

11. Please select the option that best describes the availability of supplies used in the conduct of pediatric and adult congenital cardiac catheterizations at your hospital/medical center.

Cardiac Catheterization Supplies

	Always	Sometimes	Never
Exchange length guidewires	○	○	○
Torque control guidewires	○	○	○
Coronary guidewires (< 0.014 inches/0.3556 mm thick)	○	○	○
Short introducer sheaths	○	○	○
Long introducer sheaths	○	○	○
Thermodilution catheters	○	○	○
Swan-Ganz catheters	○	○	○
Standard angioplasty balloons (burst pressure ≤10 atm)	○	○	○
High pressure angioplasty balloons (burst pressure between 11 and 20 atm)	○	○	○
Ultra-high pressure angioplasty balloons (burst pressure >20 atm)	○	○	○
Small covered intravascular stents (≤0.25 inch diameter)	○	○	○
Large covered intravascular stents (>0.25 inch diameter)	○	○	○
Bare metal intravascular stents	○	○	○
Coronary stents	○	○	○
PDA closure devices	○	○	○
ASD closure devices	○	○	○
VSD closure devices	○	○	○

12. How often do you re-sterilize and re-use items in the catheterization laboratory that are typically for single-use (i.e., as indicated by the manufacturer)?

○ Never ○ Rarely ○ Sometimes ○ Often ○ Always

Please specify which equipment is re-sterilized and re-used:

13. How often do you use items in the catheterization laboratory beyond their manufacturer recommended expiration dates?

◯ Never ◯ Rarely ◯ Sometimes ◯ Often ◯ Always

Please specify which equipment are utilized beyond their manufacturer recommended expiration date:

14. Do you have access to and staffing for general anesthesia during catheterization cases if needed?

◯ Yes
◯ No

If yes, what percentage (%) of pediatric and congenital cardiac catheterizations were performed under general anesthesia in 2015?

____

If yes, what percentage (%) of pediatric and adult congenital cardiac catheterizations were performed under conscious deep sedation in 2015?

____

15. How do you store catheterization patient images for review, clinician access, and archiving?

◯ Images are stored in digital format in a patient database maintained by the hospital/medical center
◯ Images are stored on film in a database maintained by the hospital/medical center
◯ Images are printed and stored in the clinical chart
◯ Electronic copies of the images are provided to patients for self-storing
◯ Printed copies of images are provided to patients for self-storing
◯ Catheterization lab images are not stored but only viewed in real time
◯ Other

If other, please describe:

16. How long on average does it take to retrieve stored patient images, if required?

◯ Less than one hour
◯ Less than 4 hours
◯ Less than 24 hours
◯ Other

If other, please describe:

17. Do you have any limitations related to cardiac catheterization at your hospital/medical center that restrict the number and complexity of pediatric cardiac and adult congenital cardiac operations that your team can perform?

◯ Yes
◯ No

If yes, please describe:

Section 3. Intensive Care Unit (ICU)

SECTION 3. INTENSIVE CARE UNIT (ICU)

1. Please select the option that best describes your ICU following cardiac surgery and/or interventional catheterization for the majority of pediatric cardiac patients.

◯ The ICU is dedicated for pediatric cardiac patients requiring critical care.
◯ The ICU is shared by pediatric and adult (congenital and acquired) cardiac patients.
◯ The ICU is used for the majority of pediatric cardiac patients requiring critical care.
◯ The ICU is used for all patients requiring critical care.
◯ Other

If other, please describe:

2. Please select the option that best describes your ICU following cardiac surgery and/or interventional catheterization for the majority of adult congenital heart disease patients.

◯ Adult congenital heart disease patients go to pediatric ICU and not the adult acquired ICU.
◯ Adult congenital heart disease patients go to acquired adult ICU and not the pediatric ICU.
◯ The ICU is used for all adult cardiac and non-cardiac patients requiring critical care.
◯ The ICU is used for all patients requiring critical care.
◯ Other

If other, please describe:

3. How far is the ICU from the operating room(s)?

◯ On the same floor
◯ In the same building but on a different floor
◯ In a different building
◯ Other

If other, please describe:

4. Regarding respiratory management during the transfer of the majority of patients from the operating room to the ICU, please select the option that best applies.

◯ Postoperative patients are connected to a ventilator during transport
◯ Post-operative patients are hand bagged during transport
◯ Post-operative patients are only transported after extubation in the operating room
◯ Other

If other, please describe:

5. Is the attending cardiac surgeon always present during patient transportation from the operating room to the ICU?

◯ Yes
◯ No

6. If no, please specify who leads the patient transportation team from the operating room to the ICU

7. Is there a formal multi-disciplinary handover of post-operative patients on arrival to the ICU?

◯ Yes
◯ No

8. Please indicate who participates in the handover. Select all that apply.

☐ Operating room nurses
☐ ICU nurses
☐ Intensive care physicians
☐ Surgeons
☐ Anesthesiologists
☐ Respiratory therapists
☐ Other

If other, please describe:

9. Who is the primary person, at the attending/consultant level, responsible for the day-to-day care delivery for the majority of your congenital cardiac patients in the ICU?

◯ General intensivist
◯ Cardiac intensivist
◯ Cardiac surgeon
◯ Interventional cardiologist
◯ Other

If other, please describe:

10. Where are the majority of neonates requiring cardiac critical care cared for pre-operatively?

◯ In a separate neonatal ICU (NICU).
◯ In the same ICU for post-operative or post-catheterization pediatric cardiac patients.
◯ Other

If other, please describe:

11. Does the ICU have ventilators for neonate patients?

◯ Yes
◯ No

12. Does the ICU have high frequency oscillators for neonates?

◯ Yes
◯ No

13. Does the ICU have incubator beds?

◯ Yes
◯ No

14. Does the ICU have warming tables?

◯ Yes
◯ No

15. What is the ICU bed capacity (i.e number of staffed beds) for pediatric cardiac and adult congenital cardiac patients?

____

16. On a typical weekday, how many pediatric cardiac and adult congenital cardiac patients are admitted to your ICU?

____

17. Are formal rounds conducted at least once daily in the ICU?

◯ Yes
◯ No

If no, please describe how often formal rounds are conducted:

Who attends formal rounds in the ICU? Please check all that apply.

☐ Pediatric and congenital cardiac intensivist
☐ Pediatric and congenital cardiac surgeon
☐ Pediatric and congenital cardiologist
☐ Critical care nurse
☐ Respiratory therapist
☐ Nutritionist
☐ Other

If other, please describe:

18. Please specify how often you monitor the following parameters in your ICU:

	Every hour	Every 4-6 hours	Never
Heart rate	○	○	○
Pulse oximetry	○	○	○
End tidal CO2	○	○	○
Invasive blood pressure (arterial line)	○	○	○
Central venous pressure	○	○	○
Serum lactate levels	○	○	○
Urine output	○	○	○
Other	○	○	○

If other, please describe:

19. Does the ICU have a dedicated code cart for storage of emergency medication and supplies in case of a cardiac or respiratory emergency?

○ Yes
○ No

Is this code cart fully stocked with all the necessary medications and supplies required in emergency situations?

○ Yes, the code cart is always fully stocked
○ The code cart is sometimes fully stocked
○ No, the code cart is not fully stocked

If not fully stocked, please describe what is typically missing:

Are the medications on the code cart in pediatric formulation such that in an emergency it would be easy to administer the appropriate pediatric doses?

○ Yes, all of the medications are in pediatric formulation
○ Yes, some of the medications are in pediatric formulation
○ No, the medications are not in pediatric formulation

If medications not in pediactric formulation, please describe:

20. Does the ICU have dedicated sterile emergency instruments/tools (e.g. sternal retractor) in case of the need for ICU procedures?

◯ Yes
◯ No

If no, what instruments are used for emergency ICU procedures?

21. Does your hospital/medical center have an ECMO program?

◯ Yes
◯ No

If yes, how many ECMO machines available?

____

Please describe the name, model, and year of manufacture of your ECMO machine(s):

Does your hospital/medical center have a dedicated ECMO team?

◯ Yes
◯ No

If yes, who is included on your team?

☐ Surgeon(s)
☐ Anesthesiologist(s)
☐ ECMO biomedical technician(s)
☐ ECMO respiratory therapist(s)
☐ ECMO nurse(s)
☐ Other

If other, please describe:

22. For patients in advanced heart failure or those who fail to wean from ECMO, please indicate what options are available for circulatory support:

	Yes	No
Heart Transplantation Program	○	○
Ventricular Assist Decive (VAD) Implantation	○	○
Other	○	○

If other, please describe:

23. Do you have any limitations related to your ICU that restrict the number and complexity of postoperative pediatric cardiac and adult congenital cardiac patients for whom you can provide critical care?

○ Yes
○ No

If yes, please describe:

Section 4. Step-down and Regular Ward

SECTION 4. STEP-DOWN AND REGULAR WARD(S)

1. Upon discharge from the ICU, do your pediatric cardiac and adult congenital cardiac patients get admitted to an intermediate care ward (step-down ward(s))?

◯ Yes, patients get admitted to an intermediate care (step-down) ward(s).
◯ No, patients are admitted directly to the regular ward(s) from the ICU.
◯ Other

If other, please describe:

How many staffed beds are available in your step-down ward(s)?

____

How many staffed beds are available in your regular ward(s)?

____

On a typical day, how many pediatric cardiac and adult congenital cardiac patients are admitted to your intermediate care (step-down) ward(s)?

____

On a typical day, how many pediatric cardiac and adult congenital cardiac patients are admitted to your regular ward(s)?

____

2. Do you use a formal handover checklist when transferring patients out of the ICU to the step-down or regular ward?

◯ Yes
◯ No

If yes, which of the following items are part of your handover checklist when transferring a patient out of the ICU? Please check all that apply.

☐ Medication list, doses, and routes
☐ Indwelling lines and tubes
☐ Allergies, if any
☐ Current diet
☐ Significant ICU events

3. Do you have any limitations related to your ward(s) that restrict the number and complexity of pediatric cardiac and congenital cardiac patients for whom you can provide care?

◯ Yes
◯ No

If yes, please describe:

Section 5. Cardiovascular Imaging/Radiology

SECTION 5. CARDIOVASCULAR IMAGING / RADIOLOGY

1. What type(s) of cardiovascular imaging is available at your hospital/medical center for use in pediatric patients? Check all that apply.

☐ Echocardiography
☐ Computed tomography (CT)
☐ Magnetic resonance imaging (MRI)
☐ X-ray
☐ Ultrasound
☐ Nuclear studies
☐ Other

If other, please describe:

Echocardiography

How many echocardiography machines are available?

____

Please describe the name, model, and year of manufacture of your echocardiography machine(s):

Computed tomography (CT)

Please describe name, model, and year of manufacture of your CT scanner(s):

Magnetic resonance imaging (MRI)

Please describe the name, model, and year of manufacture of your MRI scanner(s):

X-ray

Does your hospital/medical center have a portable X-ray machine(s)?

◯ Yes
◯ No

Please describe the name, model, and year of manufacture of your portable X-ray machine(s) that are available for the care of pediatric cardiac and adult congenital cardiac patients:

2. How long does it typically take to obtain an emergency echocardiogram ?

◯ Less than 1 hour after emergency identified
◯ 1 hour or more after emergency identified

3. Is your hospital/ medical center equipped to conduct echocardiogram imaging studies for infants and young children under sedation, if needed?

◯ Yes
◯ No

4. Do you have any limitations related to cardiovascular imaging/radiology that restrict the number and complexity of pediatric cardiac and adult congenital cardiac imaging studies that your team can perform?

◯ Yes
◯ No

If yes, please describe:

Section 6. Outpatient and Postoperative Follow-up

SECTION 6. OUTPATIENT AND POSTOPERATIVE FOLLOW-UP

1. Please describe how often prenatal screening is conducted for pregnant mothers in your region/catchment area for the detection of congenital heart defects.

○ Never ○ Rarely ○ Sometimes ○ Often ○ Always

Once congenital heart defects are identified on prenatal screening, how often is fetal echocardiography conducted?

○ Never ○ Rarely ○ Sometimes ○ Often ○ Always

2. Please describe how often newborn children in your region are screened for congenital heart defects at birth.

○ Never ○ Rarely ○ Sometimes ○ Often ○ Always

How is newborn screening for congenital heart disease performed in your region? Check all that apply.

☐ Clinical examination
☐ Pulse oximetry
☐ Other

If other, please describe:

3. Does your hospital/medical center perform organized community screening of infants and children to identify cases?

○ Yes
○ No

4. What is the typical time from referral to evaluation by a pediatric cardiologist at your hospital/medical center?

○ Within 24 hours
○ Less than 1 week
○ 1-2 weeks
○ 3-4 weeks
○ More than 4 weeks
○ Other

If other, please describe:

5. What is the typical time after initial evaluation to first intervention (surgical or catheter-based) at your hospital/medical center for a critically ill patient after stabilization?

◯ 1-2 days
◯ 3-7 days
◯ Greater than 1 week
◯ Other

If other, please describe:

6. After evaluation and identification of a need for surgery, what is the typical waiting time prior to intervention for an elective surgery?

◯ Less than 1 week
◯ Less than 1-4 weeks
◯ 1-3 months
◯ Greater than 3 months
◯ Other

If other, please describe:

7. What approximate percentage of your pediatric congenital heart patients return to your hospital/medical center for follow up within the first six weeks of surgery or catheterization intervention?

◯ < 10%
◯ 10 to < 25%
◯ 25 to < 50%
◯ 50 to < 75%
◯ 75-100%

8. What approximate percentage of your pediatric congenital heart patients return to your hospital/medical center for long term ongoing care (i.e. patients receive ongoing care by your team more than a year after initial intervention)?

◯ < 10%
◯ 10 to < 25%
◯ 25 to < 50%
◯ 50 to < 75%
◯ 75-100%

9. What approximate percentage of your adult congenital heart patients return to your hospital/medical center for ongoing cardiac care?

◯ < 10%
◯ 10 to < 25%
◯ 25 to < 50%
◯ 50 to < 75%
◯ 75-100%

10. Please select the main barrier preventing patients from following up at your hospital/medical center for postoperative or post-catheterization evaluation?

- ◯ Patients live far away and are unable to travel to our hospital/medical center for follow-up due to distance and associated expenses.
- ◯ The patients' and/or patients' families do not see the need for follow-up care/evaluation.
- ◯ The physicians and care team do not see the need for follow-up care/evaluation.
- ◯ Patients follow up with their local cardiologist.
- ◯ Other

If other, please describe:

---

11. If not at your hospital/medical center, where is postoperative follow-up care/evaluation conducted?

---

12. Are there any significant challenges in how patients are referred to your hospital/medical center that limit your ability to care for patients?

- ◯ Yes
- ◯ No

If yes, please describe:

Section 7. Adult Congenital Heart Disease

SECTION 7. ADULT CONGENITAL HEART DISEASE

1. What resources do you have in place for the care of adult patients with congenital heart disease?

◯ A dedicated team for the care and follow-up of adults with congenital heart disease.
◯ No dedicated team - Adult patients with congenital heart disease are treated similarly to other adults in the general hospital.
◯ No dedicated team - Adult patients with congenital heart disease are treated similarly to pediatric patients with congenital heart disease.

2. For children diagnosed with congenital heart disease in childhood, what resources do you have for transition into long term care as adults?

◯ There is a dedicated process to transition children with congenital heart disease into long term adult care.
◯ Long term care is only arranged on a need basis.
◯ There are no arrangements for long-term care of children diagnosed with congenital heart disease into adulthood.

3. What are the common diagnoses of your adult CHD patients? Check all that apply.

☐ Atrial septal defects (ASD) / ventricular septal defects (VSD)
☐ Congenital valve defects
☐ Ebstein anomaly
☐ Coarctation of the aorta
☐ Pulmonary artery stenosis
☐ Anomalous pulmonary veins
☐ Heart transplant patients
☐ Pregnant women presenting with a new diagnosis of congenital heart defects
☐ Repaired Tetralogy of Fallot patients
☐ Fontan patients following staged single ventricle palliation.
☐ Congenital heart patients with pulmonary hypertension

If other, please describe:

Section 8. Multi-disciplinary Care

SECTION 8. MULTI-DISCIPLINARY CARE

1. What is the availability of the following non-cardiac specialists to contribute to the all-around care of your patients?

	Available within the same facility	Are in a nearby facility but consultation is readily available	Not readily available
Pediatricians	○	○	○
General Pediatric Surgery	○	○	○
Infectious Disease Specialists	○	○	○
Neurology Specialists	○	○	○
Otolaryngology Specialists	○	○	○
Pulmonary Specialists	○	○	○
Gastroenterology Specialists	○	○	○
Nephrology Specialists	○	○	○
Nutrition Specialists	○	○	○
Genetic Specialists	○	○	○

2. Do you have designated care coordinators to help arrange the multidisciplinary aspects of the medical care of your patients?

○ Yes
○ No

Please describe any details pertaining to your patient care coordination:

3. Do you have designated social workers for your patients and families?

○ Yes
○ No

Please describe any details pertaining to your social worker arraignments for your patients:

4. Do you have multidisciplinary rounds or meetings to discuss the care of complex patients requiring subspecialty input?

◯ There are regular multidisciplinary rounds or meetings to discuss complex patients.
◯ Multidisciplinary discussions are arranged as needed.
◯ There are no multidisciplinary rounds or meetings
◯ Other

Please describe any details about your multidisciplinary team discussions for complex patients:

Section 9. Manpower And Staffing

SECTION 9. MANPOWER AND STAFFING

Operating Room

Cardiac Surgeon

1. How many attending level cardiac surgeons at your hospital/medical center perform pediatric cardiac and adult congenital cardiac operations?

____

How many of your attending level cardiac surgeons exclusively perform pediatric cardiac or adult congenital cardiac operations?

____

How many of your attending level cardiac surgeons have completed formal training fellowship in congenital cardiac surgery?

____

How many of your attending level cardiac surgeons also work at an outside hospital/medical center/private practice?

____

How many years of experience does your most experienced surgeon have in pediatric surgery?

◯ Less than 2 years
◯ 2 to 4 years
◯ 5 to 10 years
◯ Greater than 10 years

How many years of experience does your least experienced surgeon have in pediatric cardiac surgery?

◯ Less than 2 years
◯ 2 years to less than 5 years
◯ 5 to 10 years
◯ Greater than 10 years

Comments:

Operating Room Nursing

2. How many operating room nurses are available at your hospital/medical center for the conduct of pediatric cardiac and adult congenital cardiac operations?

____

How many of your operating room nurses exclusively assist in pediatric cardiac or adult congenital cardiac operations?

◯ None ◯ Few ◯ Most ◯ All

How many of your operating room nurses have less than 3 years of relevant experience?

◯ None ◯ Few ◯ Most ◯ All

How many of your operating room nurses have greater than 7 years of relevant experience?

◯ None ◯ Few ◯ Most ◯ All

How many of your operating room nurses also work at an outside hospital/medical center/private practice?

◯ None ◯ Few ◯ Most ◯ All

Comments:

Cardiac Anesthesiology

3. How many anesthesiologists are available at your hospital/medical center to perform pediatric cardiac and adult congenital cardiac operations anesthesiology?

____

How many of your anesthesiologists exclusively perform pediatric cardiac or adult congenital cardiac anesthesiology?

____

How many of your anesthesiologists have completed formal training fellowship in cardiac anesthesiology?

____

How many of your anesthesiologists also work at an outside hospital/medical center/private practice?

____

How many years of experience does your most experienced cardiac anesthesiologist have in cardiac anesthesiology?

◯ Less than 2 years
◯ 2 to 4 years
◯ 5 to 10 years
◯ Greater than 10 years

How many years of experience does your least experienced cardiac anesthesiologist have in cardiac anesthesiology?

◯ Less than 2 years
◯ 2 to 4 years
◯ 5 to 10 years
◯ Greater than 10 years

Comments:

Non-Physician Anesthesia Providers

4. How many non-physician anesthesia providers are available at your hospital/medical center to perform pediatric cardiac and adult congenital cardiac operations?

____

How many of your non-physician anesthesia providers exclusively assist in pediatric cardiac or adult congenital cardiac operations?

◯ None ◯ Few ◯ Most ◯ All

How many of your non-physician anesthesia providers have less than 3 years of experience in cardiac anesthesiology?

◯ None ◯ Few ◯ Most ◯ All

How many of your non-physician anesthesia providers have greater than 7 years of experience in cardiac anesthesiology?

◯ None ◯ Few ◯ Most ◯ All

How many of your non-physician anesthesia providers also work at an outside hospital/medical center/private practice?

◯ None ◯ Few ◯ Most ◯ All

Comments:

Perfusion

5. How many perfusionists are available at your hospital/medical center for the conduct of pediatric cardiac and adult congenital cardiac operations?

____

How many of your perfusionists exclusively perform pediatric cardiac or adult congenital cardiac perfusion?

◯ None ◯ Few ◯ Most ◯ All

How many of your perfusionists have completed formal training/certification in cardiac perfusion?

◯ None ◯ Few ◯ Most ◯ All

How many of your perfusionists have less than 3 years of relevant experience?

◯ None ◯ Few ◯ Most ◯ All

How many of your perfusionists have greater than 7 years of relevant experience?

◯ None ◯ Few ◯ Most ◯ All

How many of your perfusionists also work at an outside hospital/medical center/private practice?

◯ None ◯ Few ◯ Most ◯ All

Comments:

Catheterization Laboratory

6. How many interventional cardiologists are available at your hospital/medical center participating in the conduct of pediatric cardiac and adult congenital cardiac catheterizations at your hospital/medical center?

____

How many of your interventional cardiologists exclusively perform pediatric cardiac or adult congenital cardiac catheterizations?

____

How many of your interventional cardiologists have completed formal training fellowship in interventional cardiology?

____

How many of your interventional cardiologists also work at an outside hospital/medical center/private practice?

____

How many years of experience does your most experienced interventional cardiologist have in pediatric and congenital cardiac catheterization?

◯ Less than 2 years
◯ 2 to 4 years
◯ 5 to 10 years
◯ Greater than 10 years

How many years of experience does your least experienced interventional cardiologist have in pediatric and congenital cardiac catheterization?

◯ Less than 2 years
◯ 2 to 4 years
◯ 5 to 10 years
◯ Greater than 10 years

Comments:

Catheterization Lab Nurses

7. How many catheterization laboratory nurses are available at your hospital/medical center to assist in the conduct of pediatric cardiac and adult congenital cardiac catheterizations at your hospital/medical center?

____

How many of your catheterization laboratory nurses exclusively assist in pediatric cardiac or adult congenital cardiac catheterizations?

◯ None ◯ Few ◯ Most ◯ All

How many of your catheterization laboratory nurses have less than 3 years of relevant experience?

◯ None ◯ Few ◯ Most ◯ All

How many of your catheterization laboratory nurses have greater than 7 years of relevant experience?

◯ None ◯ Few ◯ Most ◯ All

How many of your catheterization laboratory nurses also work at an outside hospital/medical center/private practice?

◯ None ◯ Few ◯ Most ◯ All

Comments:

Catheterization Lab Technicians

8. How many catheterization laboratory technicians at your hospital/medical center participate in the conduct of pediatric cardiac and adult congenital cardiac catheterizations at your hospital/medical center?

____

How many of your catheterization laboratory technicians exclusively assist in pediatric cardiac or adult congenital cardiac catheterizations?

◯ None ◯ Few ◯ Most ◯ All

How many of your catheterization laboratory technicians have less than 3 years of relevant experience?

◯ None ◯ Few ◯ Most ◯ All

How many of your catheterization laboratory technicians have greater than 7 years of relevant experience?

◯ None ◯ Few ◯ Most ◯ All

How many of your catheterization laboratory technicians also work at an outside hospital/medical center/private practice?

◯ None ◯ Few ◯ Most ◯ All

Intensive Care Unit (ICU)

Pediatric Intensive Care Physician

9. How many pediatric intensive care physicians caring for pediatric cardiac and adult congenital patients are available at your hospital/medical center?

____

How many of your pediatric intensive care physicians exclusively care for pediatric cardiac or adult congenital cardiac patients?

____

How many of your pediatric intensive care physicians have completed formal training fellowship in cardiac critical care?

____

How many of your pediatric intensive care physicians also work at an outside hospital/medical center/private practice?

____

How many years of experience does your most experienced pediatric intensive care physician have in cardiac critical care?

◯ Less than 2 years
◯ 2 to 4 years
◯ 5 to 10 years
◯ Greater than 10 years

How many years of experience does your least experienced pediatric intensive care physician have in cardiac critical care?

◯ Less than 2 years
◯ 2 to 4 years
◯ 5 to 10 years
◯ Greater than 10 years

Comments:

Intensive Care Nurses

10. How many intensive care nurses are available at your hospital/medical center for the care of pediatric cardiac and adult congenital cardiac patients?

____

How many of your intensive care nurses exclusively assist in pediatric cardiac or adult congenital cardiac cases in the intensive care unit?

◯ None ◯ Few ◯ Most ◯ All

How many of your intensive care nurses have less than 3 years of relevant experience?

◯ None ◯ Few ◯ Most ◯ All

How many of your intensive care nurses have greater than 7 years of relevant experience?

◯ None ◯ Few ◯ Most ◯ All

How many of your intensive care nurses also work at an outside hospital/medical center/private practice?

◯ None ◯ Few ◯ Most ◯ All

Comments:

Non-Physician Clinical Providers

Physician Assistants (PAs), Nurse Practitioners, and Similar Personnel

11. What type of non-physician clinical providers participate in the care of pediatric cardiac and adult congenital cardiac patients in your intensive care unit? Please check all that apply.

☐ Physician Assistants
☐ Advanced Nurses or Nurse Practitioners
☐ Other licensed professionals
☐ Other ancillary professionals
☐ Other

If other, please describe:

12. How many non-physician clinical providers (physician assistants (PAs), nurse practitioners, and similar personnel) participating in the care of pediatric cardiac and adult congenital cardiac patients are available in your intensive care unit?

____

How many non-physician clinical providers (physician assistants (PAs), nurse practitioners, and similar personnel) exclusively assist in the care of pediatric cardiac or adult congenital cardiac patients?

◯ None ◯ Few ◯ Most ◯ All

How many of the non-physician clinical providers (physician assistants (PAs), nurse practitioners, and similar personnel) have less than 3 years of relevant experience?

◯ None ◯ Few ◯ Most ◯ All

How many of the non-physician clinical providers (physician assistants (PAs), nurse practitioners, and similar personnel) have greater than 7 years of relevant experience?

◯ None ◯ Few ◯ Most ◯ All

How many of the non-physician clinical providers (physician assistants (PAs), nurse practitioners, and similar personnel) also work at an outside hospital/medical center/private practice?

◯ None ◯ Few ◯ Most ◯ All

Nurse Aides/Nursing Technicians

13. How many nurse aides/nursing technicians participating in the care of pediatric cardiac and adult congenital cardiac patients are available at your intensive care unit?

____

How many of your nurse aides/nursing technicians exclusively assist in the care of pediatric cardiac or adult congenital cardiac patients?

◯ None ◯ Few ◯ Most ◯ All

How many of the nurse aides/nursing technicians have less than 3 years of relevant experience?

◯ None ◯ Few ◯ Most ◯ All

How many of the nurse aides/nursing technicians have greater than 7 years of relevant experience?

◯ None ◯ Few ◯ Most ◯ All

How many of the nurse aides/nursing technicians also work at an outside hospital/medical center/private practice?

◯ None ◯ Few ◯ Most ◯ All

Comments:

14. What is the day time provider-to-patient ratio of nurses and nurse aides/nursing technicians in the intensive care unit where your pediatric cardiac and adult congenital cardiac patients are cared for?

	1:1	1:2	1:3	Other
Nurse	◯	◯	◯	◯
Nurse Aide/Nursing Technician	◯	◯	◯	◯

If other provider-to-patient ratio, please specify (Nurse):

____

If other provider-to-patient ratio, please specify (Nurse Aide/Nursing Technician):

____

Please provide any comments you may have regarding provider-to-patient ratio for non-physician pediatric intensive care providers during the day:

15. What is the night time provider-to-patient ratio of nurses and nurse aides/nursing technicians in the intensive care unit where your pediatric cardiac and adult congenital cardiac patients are cared for?

	1:1	1:2	1:3	Other
Nurse	◯	◯	◯	◯
Nurse Aide/Nursing Technician	◯	◯	◯	◯

If other provider-to-patient ratio, please specify (Nurse):

____

If other provider-to-patient ratio, please specify (Nurse Aide/Nursing Technicians):

____

Please provide any comments you may have regarding provider-to-patient ratio for non-physician pediatric intensive care providers during the night:

Respiratory Therapists

16. How many respiratory therapists caring for pediatric cardiac and adult congenital cardiac patients are available at your hospital/medical center?

____

How many of your respiratory therapists exclusively assist in the care of pediatric cardiac or adult congenital cardiac patients?

◯ None ◯ Few ◯ Most ◯ All

How many of your respiratory therapists have less than 3 years of relevant experience?

◯ None ◯ Few ◯ Most ◯ All

How many of your respiratory therapists have greater than 7 years of relevant experience?

◯ None ◯ Few ◯ Most ◯ All

How many of your respiratory therapists also work at an outside hospital/medical center/private practice?

◯ None ◯ Few ◯ Most ◯ All

Comments:

Cardiologists

17. How many cardiologists are employed at your hospital/medical care center for the care of pediatric cardiac and adult congenital cardiac patients?

____

How many of your cardiologists exclusively care for pediatric and congenital cardiac patients?

____

How many of your cardiologists completed formal training fellowship in pediatric cardiology?

____

How many of your cardiologists also work at an outside hospital/medical center/private practice?

____

How many years of experience does your most experienced cardiologist have in pediatric and adult congenital cardiac care?

◯ Less than 2 years
◯ 2 to 4 years
◯ 5 to 10 years
◯ Greater than 10 years

How many years of experience does your least experienced cardiologist have in pediatric and adult congenital cardiac care?

◯ Less than 2 years
◯ 2 to 4 years
◯ 5 to 10 years
◯ Greater than 10 years

Comments:

Nursing on the step-down or regular wards

18. How many nurses caring for pediatric cardiac and adult congenital patients in the step-down/intermediate care or regular wards are available at your hospital/medical center?

____

How many of your nurses in the step-down/intermediate care or regular wards exclusively care for pediatric cardiac and adult congenital cardiac patients?

◯ None ◯ Few ◯ Most ◯ All

How many of your nurses in the step-down/intermediate care or regular wards have less than 3 years of relevant experience?

◯ None ◯ Few ◯ Most ◯ All

How many of your nurses in the step-down/intermediate care or regular wards have greater than 7 years of relevant experience?

◯ None ◯ Few ◯ Most ◯ All

How many of your nurses in the step-down/intermediate care or regular wards also work at an outside hospital/medical center/private practice?

◯ None ◯ Few ◯ Most ◯ All

Non-Physician Clinical Providers (Physician Assistants (PAs), Nurse Practitioners, and Similar Personnel)

19. What type of non-physician clinical providers participate in the care of pediatric cardiac and adult congenital patients in your step-down/intermediate care or regular wards?

☐ Physician Assistants
☐ Advanced Nurses or Nurse Practitioners
☐ Other licensed professionals
☐ Other ancillary professionals
☐ Other

If other, please describe:

20. How many non-physician clinical providers (physician assistants (PAs), nurse practitioners, and similar personnel) in the step-down and/or regular ward caring for pediatric cardiac and adult congenital cardiac patients are available at your hospital/medical center?

____

How many of your non-physician clinical providers (physician assistants (PAs), nurse practitioners, and similar personnel) in the step-down and/or regular ward exclusively care care for pediatric cardiac and adult congenital cardiac patients?

◯ None ◯ Few ◯ Most ◯ All

How many of your non-physician clinical providers (physician assistants (PAs), nurse practitioners, and similar personnel) in the step-down and/or regular ward have less than 3 years of relevant experience?

◯ None ◯ Few ◯ Most ◯ All

How many of your non-physician clinical providers (physician assistants (PAs), nurse practitioners, and similar personnel) in the step-down and/or regular ward have greater than 7 years of relevant experience?

◯ None ◯ Few ◯ Most ◯ All

How many of your non-physician clinical providers (physician assistants (PAs), nurse practitioners, and similar personnel) in the step-down and/or regular ward also work at an outside hospital/medical center/private practice?

◯ None ◯ Few ◯ Most ◯ All

Comments:

21. What is the typical provider to patient ratio for nurses and nursing aides/nursing technicians on the intermediate care/ step-down ward?

	1:1	1:2	1:3	Other ratio
Nurse	◯	◯	◯	◯
Nurse Aide/Nursing Technician	◯	◯	◯	◯

If other provider-to-patient ratio, please specify (Nurse):

____

If other provider-to-patient ratio, please specify (Nurse Aide/Nursing Technician):

____

22. What is your typical provider-to-patient ratio for nurses and nursing technicians in your regular ward?

	1:1	1:2	1:3	Other
Nurse	◯	◯	◯	◯
Nurse Aide/Nursing Technician	◯	◯	◯	◯

If other provider-to-patient ratio, please specify (Nurse):

____

If other provider-to-patient ratio, please specify (Nurse Aide/Nursing Technician):

____

Pediatricians

23. How many pediatricians are available to care for pediatric cardiac patients?

____

Comments:

Visiting teams

24. Do you have a program where visiting teams come to your hospital and contribute to patient care for pediatric cardiac and adult congenital cardiac surgery?

◯ Yes, we currently have a program
◯ No, but we had a program in the past
◯ No, we do not have a program

What is the approximate number of times that teams visit per year?

____

How long ago did you have a visiting team program?

◯ Less than or equal to 3 years
◯ More than 3 years ago

Please indicate what professionals regularly visit your health care facility to contribute to your patient care? Check all the apply.

☐ Cardiac surgeons
☐ Interventional cardiologists
☐ Cardiologists
☐ Perfusionists
☐ Catheterization lab technicians
☐ Other

If other, please describe:

What is the current annual percentage of total surgical procedures that the above visiting staff contribute?

◯ < 30%
◯ 30-70%
◯ >70%

What is the current annual percentage of total cardiac catheterizations that the above visiting staff contribute?

◯ < 30%
◯ 30-70%
◯ >70%

Where do the visiting teams in your program come from? Please check all that apply.

☐ Within the same country
☐ Within the region
☐ Outside the region

25. Do you have any limitations related to manpower and staffing at your hospital/medical center that restrict the number and complexity of pediatric cardiac and adult congenital cardiac operations that your team can perform?

◯ Yes
◯ No

If yes, please clarify:

Section 10. Administrative Characteristics

SECTION 10. ADMINISTRATIVE CHARACTERISTICS

Cardiovascular Program Catchment Area

1. Is your cardiovascular program the only program in your country available for the surgical management of patients with pediatric cardiac and adult congenital cardiac disease?

◯ Yes
◯ No

If no, how many other cardiovascular programs are there available in your country for the surgical management of patients with pediatric cardiac and adult congenital cardiac disease?

____

2. Please indicate the approximate percentage of pediatric cardiac and adult congenital cardiac patients that undergo surgical evaluation and treatment at your hospital/medical center who travel the following distances to get to your center.

< =50 km (%) __________________________________

51-200 km (%) __________________________________

201-500 km (%) __________________________________

>500 km (%) __________________________________

3. Is your cardiovascular program the only program in your country available for the conduct of interventional or diagnostic catheterization for patients with pediatric cardiac and adult congenital cardiac disease?

◯ Yes
◯ No

If no, how many other cardiovascular programs are there available in your country for interventional or diagnostic catheterization for patients with pediatric cardiac and adult congenital cardiac disease?

____

4. Please indicate the approximate percentage of patients that undergo interventional or diagnostic cardiac catheterization at your hospital/medical center who travel the following distances to get to your hospital/medical center.

< =50 km (%) ________________________

51-200 km (%) ________________________

201-500 km (%) ________________________

>500 km (%) ________________________

Financial Organization of Cardiovascular Program

5. Please indicate which option best describes the financial organization of your hospital/medical center.

- ◯ Public hospital/medical center (i.e. the majority of funds for hospital operations are from public/government subsidies)
- ◯ Non-governmental hospital/medical center (i.e. the majority of funds for hospital operations are from non-governmental orgnizations, donations, or endowments)
- ◯ Private hospital/medical center (i.e. the majority of funds for hospital operations are from fee-for-service charges)
- ◯ Other

If other, please describe:

6. Of the patients seen at your hospital/medical center in 2015, please indicate the approximate percentages supported by the following:

Self-pay (%): ________________________

Private health insurance (%): ________________________

Government-sponsored insurance (%): ________________________

Charitable donations (%): ________________________

Other (%): ________________________

If other, please describe:

7. For patients with private health insurance, what percentage of hospital discharges do most patients pay out of pocket?

◯ Less than 10%
◯ 10% - 50%
◯ Greater than 50%
◯ Not Applicable (N/A)

8. For patients with government-sponsored insurance, what percentage of hospital discharges do most patients pay out of pocket?

◯ Less than 10%
◯ 10% - 50%
◯ Greater than 50%
◯ Not Applicable (N/A)

9. For patients utilizing charitable donations, what percentage of hospital discharges do most patients pay out of pocket?

◯ Less than 10%
◯ 10% - 50%
◯ Greater than 50%
◯ Not Applicable (N/A)

10. For each of the following procedures, please indicate the typical cost per patient encounter:

Select your currency:

◯ Afghan afghani (AFN)
◯ Argentine peso (ARS)
◯ Bangladeshi taka (BDT)
◯ Brazilian real (BRL) ◯ Bulgarian lev (BGN) ◯ Chinese Ren Ming Bi (RMB)
◯ Chinese yuan (CNY) ◯ Colombian peso (COP) ◯ Costa Rican colón (CRC)
◯ Europe Union Euro (EUR)
◯ Georgian lari (GEL) ◯ Guatemalan quetzal (GTQ) ◯ Indian rupee (INR)
◯ Malaysian ringgit (MYR)
◯ Mexican peso (MXN)
◯ Nicaraguan córdoba (NIO)
◯ Pakistani rupee (PKR)
◯ Peruvian nuevo sol (PEN)
◯ Russian ruble (RUB) ◯ Serbian dinar (RSD) ◯ Ugandan shilling (UGX)
◯ Ukrainian hryvnia (UAH)
◯ United States dollar (USD)
◯ Vietnamese dong (VND)
◯ Other

If other, please specify:

____

Cost of one diagnostic echocardiography:	________________________
Cost of one diagnostic catheterization, including cost for staff time and catheterization laboratory time:	________________________
Cost of one uncomplicated interventional catheterization, including cost for staff time and recovery, but excluding the cost of implantable medical devices:	________________________
Cost of one uncomplicated closed surgical operation (e.g., PDA), including cost for staff time and operating room time, but excluding the cost of implantable medical devices:	________________________
Cost of one uncomplicated open heart surgical operation (i.e., RACHS-1 risk category 1-3), including cost for staff time and operating room time, but excluding the cost of implantable medical devices:	________________________
Cost of a routine, non-complicated ICU stay (2-3 days):	________________________
Cost of a one-week hospital stay (not including ICU):	________________________
Cost of one initial outpatient evaluation, including laboratory testing and electrocardiography, but excluding the cost of echocardiography or radiographic imaging:	________________________
Cost of one postoperative follow-up outpatient evaluation, including laboratory testing and electrocardiography, but excluding the cost of echocardiography or radiographic imaging:	________________________

Quality Improvement

11. Is your hospital/medical center accredited by the Joint Commission International (JCI), International Organization for Standardization (ISO), or a similar independent certification organization?

○ Yes, my hospital/medical center is accredited by the JCI.
○ Yes, my hospital/medical center is accredited by the ISO.
○ Yes, my hospital/medical center is accredited by another organization.
○ No, my hospital/medical center is not accredited.

If accredited by another organization, please specify: ________________________

Infection Control

12. Does your hospital/medical center have a formal infection control program?

◯ Yes
◯ No

If yes, are there infection control personnel assigned to the ICU?

◯ Yes
◯ No

If no, please describe:

13. Is there active surveillance for bacterial colonization in your ICU?

◯ Yes
◯ No

If yes, please describe:

14. Is there active surveillance for postoperative infection?

◯ Yes
◯ No

If yes, please check all that apply where surveillance is active:

☐ Catheter-associated urinary tract infections
☐ Central line-associated bloodstream infections
☐ Ventilator-associated infections
☐ Surgical site infections
☐ Other

If other, please describe:

15. Please indicate what resources you have for cleaning the general hospital spaces and wards, including the ICU.

◯ A dedicated environmental service is responsible for the daily cleaning of hospital spaces.
◯ Daily cleaning of hospital spaces are the responsibility of the hospital staff that work in those areas.
◯ Cleaning of the hospital spaces is contracted to and outside company.
◯ Hospital spaces are cleaned as needed and there is no formal cleaning arrangement.
◯ Other

If other, please describe:

Training

16. Is your hospital/medical center affiliated with a medical school?

◯ Yes
◯ No

17. Is your hospital/medical center affiliated with a nursing school?

◯ Yes
◯ No

18. Does your hospital/medical center have a formal training program for cardiac surgery?

◯ Yes
◯ No

Patient Demographics

19. How many surgeries did your team perform for congenital heart conditions among pediatric patients in 2015?

____

20. How many surgeries did your team perform for acquired heart conditions among pediatric patients (such as rheumatic heart disease) in 2015?

____

21. Do you operate on adults (greater than 18 years) with congenital heart disease?

◯ Yes
◯ No

If yes, approximately how many surgeries were performed on adults with congenital heart disease in 2015?

____

Of these approximately how many surgeries were performed as the initial surgical intervention for a congenital condition?

____

Approximately how many surgeries were performed as follow-up or repeat interventions for congenital conditions that were initially treated surgically during childhood?

____

22. Please indicate what factors limit the number or complexity of pediatric cardiac and adult congenital heart surgeries that you are able to perform at your center:

23. How many cardiac catheterizations were performed in your congenital heart program in 2015?

____

24. How many pediatric interventional cardiac catheterizations were performed for patients with congenital heart disease in your congenital heart program in 2015?

____

25. Please indicate what factors limit the number or complexity of congenital interventional or diagnostic catheterizations that you are able to perform at your center:

Maintenance and Repair of Equipment

26. Who is responsible for the maintenance and repair of your cardiovascular equipment?

◯ A dedicated biomedical engineering team for cardiovascular equipment.
◯ A general biomedical engineering team all units in the hospital.
◯ An external biomedical engineering team as needed for a fee.
◯ The equipment manufacturer as needed.
◯ No specific arrangement is available for the maintenance and repair of cardiovascular equipment.
◯ Other

If other, please describe:

27. Is your cardiovascular equipment maintained according to manufacturer specifications?

◯ Never ◯ Rarely ◯ Sometimes ◯ Often ◯ Always

28. Is your operating room equipment, including anesthesia equipment and bypass machine, insured or maintained on a manufacturer warranty?

◯ Yes
◯ No

If no, please describe:

29. Is your ICU equipment, including respirators and monitors, insured or maintained on a manufacturer warranty?

◯ Yes
◯ No

If no, please describe:

General Infrastructure

30. Is there a back-up plan in place in the event of a water shortage at your hospital/medical center?

◯ Yes
◯ No

If yes, please describe:

31. What is the primary power supply for your equipment and lighting?

◯ National electric grid
◯ Power is supplied on site by a fuel-powered generator
◯ Power is supplied on site by solar generation
◯ Other

If other, please describe:

32. Is there access to a back-up power source in the event of power failure at your hospital/medical center?

◯ Yes
◯ No

If yes, please describe:

If yes, are all areas of the hospital covered by the backup power supply?

◯ Yes
◯ No

If no, please indicate if the following areas are covered by backup power:

☐ ICU
☐ Operating room
☐ Catheterization lab
☐ Imaging suite

33. Are surge protectors installed to protect critical equipment in the event of an unanticipated power surge?

◯ Yes
◯ No

34. How does your hospital/medical center dispose of hazardous medical waste?

◯ Medical waste is disposed by a centralized government agency.
◯ Medical waste is disposed by a private contractor for fee from the hospital.
◯ Medical waste is disposed by an onsite incinerator and sharps disposal system.
◯ Other

If other, please describe:

Medical Records and Communication

35. Please describe your medical records system:

◯ Electronic medical record system
◯ Paper chart-based medical record system
◯ Mixed system of both electronic and paper chart-based records
◯ Other

If other, please describe:

36. Please indicate the primary system used by providers to order patient treatments, interventions, and diagnostic tests.

◯ Electronic ordering system
◯ Paper ordering system - providers write and sign orders on a designated paper or document orders in a patient's paper chart.
◯ Other

If other, please describe:

37. Please rate the level of impact that the following forms of possible additional support to your care team or to your adult CHD patients and their families would have on the care for adult CHD patients (no impact , some positive impact, high positive impact)

Possible Additional Support to Care Team

	No Impact	Some Impact	High Impact
On-line ACHD webinars	◯	◯	◯
Access to journal articles on ACHD topics	◯	◯	◯
Written materials on ACHD topics	◯	◯	◯
In-person training visits from ACHD experts	◯	◯	◯
Site visits of your staff to ACHD tertiary care centers	◯	◯	◯
On-going partnering with ACHD tertiary care centers	◯	◯	◯
Access to telemedicine resources from ACHD tertiary care centers	◯	◯	◯
Written material for patients on life-long care needs	◯	◯	◯
In-person educational events for patients on life-long care needs	◯	◯	◯
Peer-to-peer education on life-long care needs for patients	◯	◯	◯
Support groups for families	◯	◯	◯
Support groups for ACHD patients	◯	◯	◯
Other	◯	◯	◯

Please indicate any other form of support:

38. If the IQIC (or other partner organization) could provide additional support to your program, what type of support would be most helpful to your team?
